# Structural divergence and phylogenetic relationships of *Ajania* (Asteraceae) from plastomes and ETS

**DOI:** 10.1186/s12864-023-09716-4

**Published:** 2023-10-10

**Authors:** Jingya Yu, Yun Han, Hao Xu, Shuang Han, Xiaoping Li, Yu Niu, Shilong Chen, Faqi Zhang

**Affiliations:** 1 Key Laboratory of Adaptation and Evolution of Plateau Biota, Northwest Institute of Plateau Biology & Institute of Sanjiangyuan National Park, Chinese Academy of Sciences, Xining, 810008 China; 2 University of Chinese Academy of Sciences, Beijing, 100039 China; 3 Qinghai Provincial Key Laboratory of Crop Molecular Breeding, Xining, 810008 China

**Keywords:** *Ajania*, Phylogeny, Plastome, ETS, Molecular markers

## Abstract

**Background:**

*Ajania* Poljakov, an Asteraceae family member, grows mostly in Asia’s arid and semi-desert areas and is a significant commercial and decorative plant. Nevertheless, the genus’ classification has been disputed, and the evolutionary connections within the genus have not been thoroughly defined. Hence, we sequenced and analyzed *Ajania*’s plastid genomes and combined them with ETS data to assess their phylogenetic relationships.

**Results:**

We obtained a total of six new *Ajania* plastid genomes and nine ETS sequences. The whole plastome lengths of the six species sampled ranged from 151,002 bp to 151,115 bp, showing conserved structures. Combined with publicly available data from GenBank, we constructed six datasets to reconstruct the phylogenetic relationships, detecting nucleoplasmic clashes. Our results reveal the affinities of *Artemisia*, *Chrysanthemum* and *Stilpnolepis* to *Ajania* and validate the early taxonomy reclassification. Some of the plastid genes with low phylogenetic information and gene trees with topological differences may have contributed to the ambiguous phylogenetic results of *Ajania*. There is extensive evolutionary rate heterogeneity in plastid genes. The *psbH* and *ycf2* genes, which are involved in photosynthesis and ATP transport, are under selective pressure. Plastomes from *Ajania* species diverged, and structural aspects of plastomes may indicate some of the real evolutionary connections. We suggest the *ycf1* gene as a viable plastid DNA barcode because it has significant nucleotide diversity and better reflects evolutionary connections.

**Conclusion:**

Our findings validate the early *Ajania* taxonomy reclassification and show evolutionary rate heterogeneity, genetic variety, and phylogenetic heterogeneity of plastid genes. This research might provide new insights into the taxonomy and evolution of *Ajania*, as well as provide useful information for germplasm innovation and genetic enhancement in horticultural species.

**Supplementary Information:**

The online version contains supplementary material available at 10.1186/s12864-023-09716-4.

## Background

*Ajania* Poljakov comprises predominantly perennial herbs, semi-shrubs, or shrubs under Asteraceae, containing approximately 30 taxa, mainly distributed in desert and semi-desert regions of Asia [1]. The majority of *Ajania* species possess significant commercial value and are frequently employed as fungicides, insecticides, and ornamental plants [2, 3]. The interbreeding compatibility between *Ajania* and *Chrysanthemum* has resulted in the widespread utilization of the genus for the enhancement of decorative blooms [4, 5]. This enhancement must be based on taxonomy rather than being viewed as a precursor to trait introgression [4].

*Ajania* was formerly classified in *Artemisia*, but Poljakov [1] separated the genus from *Artemisia* based on the spreading corolla lobes, all florets being fertile, and corymbose synflorescence. According to Tzvelev [6], *Ajania* and *Chrysanthemum* are phylogenetically related, originating from a shared ancestor with radiating capitula [7]. *Ajania* was initially classified as a member of *Chrysanthemum* based on morphological evidence, as well as examination of the internal transcribed spacer (ITS) area and external transcribed spacer (EST) region [8–10]. Several molecular phylogenetic analyses have been conducted in order to elucidate the taxonomic distinction between *Ajania* and *Chrysanthemum* [11–14]. However, the outcomes consistently demonstrated that both genera are polyphyletic taxa and failed to effectively differentiate between *Ajania* and *Chrysanthemum*.

Muldashev (1981) separated *Phaeostigma* from *Ajania* as a distinct genus based on brownish style-branches, erect corolla lobes, and *Artemisia*-type pollens. However, this taxonomic separation was not strongly supported by early molecular phylogenetic studies based on nuclear ribosomal DNA (nrDNA) and nuclear genes [10, 16, 17], but rather demonstrated nested phylogenetic relationships between *Phaeostigma* and *Ajania*. It was not until Huang et al. (2017) proposed the separation of *Phaeostigma* from *Ajania* based on an analysis of nuclear sequences, chloroplast genes, and morphological data. The genus *Ajania* has been expanded to include six species: *P. ramosum* (*A. ramosa* (Chang) Shih), *P. purpureum* (*A. purpurea* Shih), *P. tibeticum* (*A. tibetica* (Hook. f. et Thoms. ex C. B. Clarke) Tzvel.), *P. quercifolium* (W. W. Sm.) Muldashev, *P. salicifolium* (Mattf.) Muldashev, and *P. variifolium* (*A. variifolia* (Chang) Tzvel.). Recently, several studies using low-copy nuclear loci, nrITS [13], and metabolomics [11] have demonstrated the relatively distant among *Ajania*, *Chrysanthemum*, and *Phaeostigma*, but some species of *Ajania* and *Phaeostigma* were still found to be phylogenetically nested within *Chrysanthemum*. Due to varying degrees of application, the internal phylogeny of *Ajania* has received limited attention in contemporary phylogenetic research, which mostly concentrates on *Chrysanthemum* and its evolutionary dynamics [12, 18, 19]. Comparison with the phylogeny of *Chrysanthemum*, shows that the connections within *Ajania* are still indistinct and inadequately delineated.

Currently, the primary data sources utilized in the field of phylogenetic genomics are plastomes and nuclear genomes. Plastomes possess advantageous characteristics such as uniparental inheritance, structural conservation, minimal recombination, and short sequences, making them excellent for molecular phylogenetic studies [20]. Plastid sequences have proven to be highly effective as super DNA barcodes for species identification, particularly in taxonomically challenging taxa [21], such as *Allium* L. [22], Leguminosae Juss. [23], subtribe Melocanninae of Poaceae Barnhart [24], etc. The huge number of plastid sequences offers significant insights for current taxonomic and phylogenetic studies, surpassing the limited utility of a small set of plastid or nuclear markers [25].

Current molecular phylogenetic studies have mostly focused on the separation of the genera *Chrysanthemum* and *Ajania* [16, 19], with little knowledge regarding the underlying phylogenetic relationships within *Ajania*. Furthermore, there is still a lack of large-scale datasets with rich phylogenetic signals for determining phylogenetic connections in *Ajania*. Hence, in this study, we employ plastid and ETS data to (1) update *Ajania*’s phylogenetic connections and (2) examine changes in the composition and structure of *Ajiania* plastomes. It would be helpful to resolve the phylogeny of *Ajania* and its related taxa.

## Results

### Assembly of plastomes and ETS sequences

**Fig. 1:**
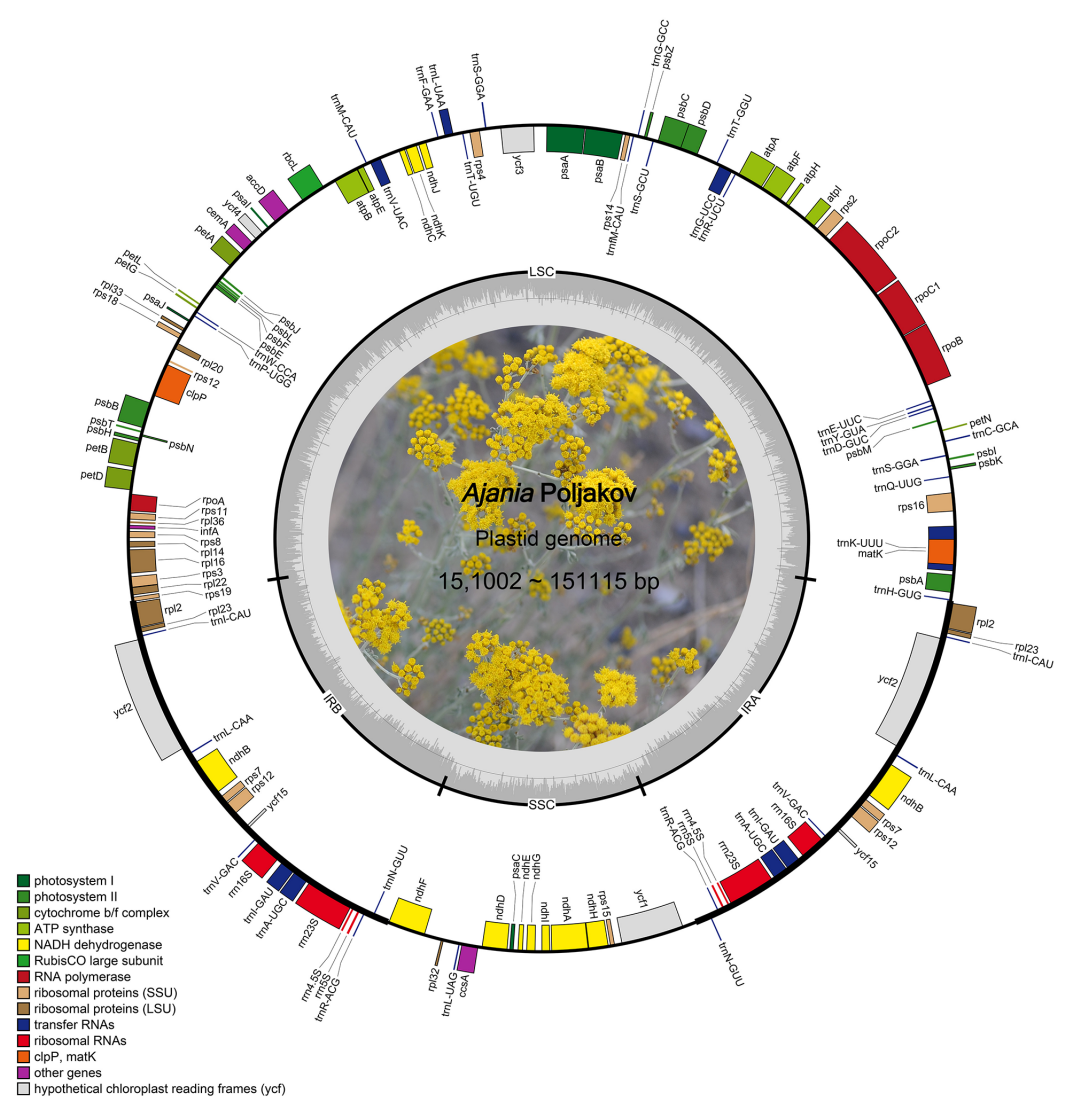
Gene map of *Ajnia* plastomes. The two gray arrows indicate the direction of gene transcription. The dashed area in the inner circle indicates the GC content of the plastome. LSC: large-single-copy; SSC: small-single-copy; IR: inverted repeat

A total of 80.6 Gb (6.8 ~ 16.0 Gb) of raw reads was obtained on the Illumina NovaSeq 2500 system. The whole plastome lengths of all species sampled ranged from 151,002 bp (*A. ramosa*) to 151,115 bp (*A. przewalskii*) and showed a tetrad structure (Fig. 1, Table S2): two inverted repeat (IR) regions ranging in length from 24,957 bp (*A. nematoloba*) to 24,967 bp (*A. ramosa*); a large single copy (LSC) region ranging in length from 82,755 bp (*A. ramosa*) to 82,856 bp (*A. przewalskii*); and a small single copy (SSC) region ranging in length from 18,313 bp (*A. ramosa*) to 18,369 bp (*A. nematoloba*). All samples encoded 132 genes, including 87 protein-coding genes, 37 tRNAs and 8 rRNAs (Table S4). These genes were arranged in a similar order between species (as exemplified in Fig. 1).

We obtained nine ETS sequences ranging in length from 840 bp (*Artemisia tangutica*) to 2,133 bp (*Brachanthemum pulvinatum*). We deposited the final annotated all plastid genomes and ETS sequences in GenBank (Table S2).

The length of each matrix after MAFFT matching and Gblock trimming of the different data was as follows: dataset I was 63,588 bp; dataset II was 42,392 bp; dataset III was 21,196 bp; dataset IV was 150,524 bp; and dataset V was 1,127 bp.

### Phylogenetic analysis of *Ajania*

**Fig. 2:**
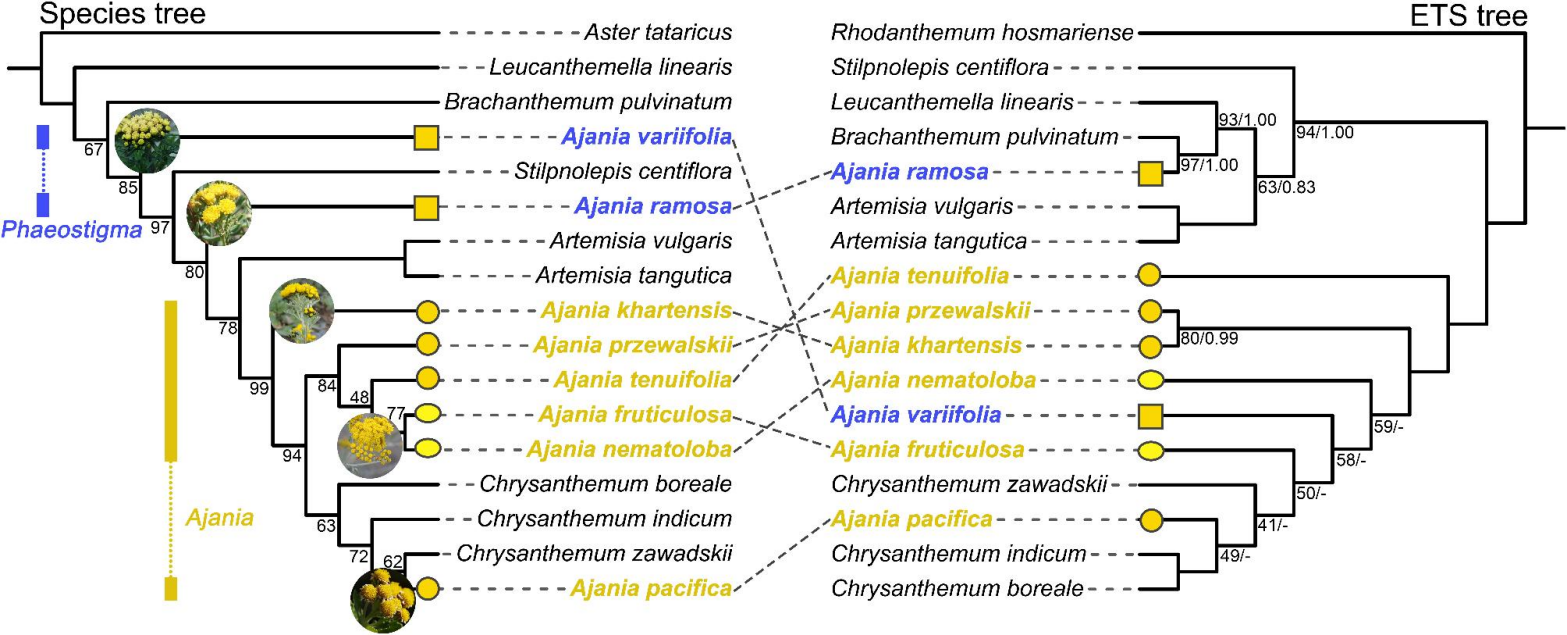
Comparison of the plastid species tree (left tree) constructed based on dataset VI (concatenated 68 CDSs) with the maximum likelihood (ML) tree (right tree) constructed based on dataset V (ETS sequences). The values associated with branches are ML bootstrap values and Bayesian posterior probabilities. Nodes of species tree with no numbers indicate 100% bootstrap. Nodes of ETS tree with no numbers indicate 100% bootstrap support and 1.0 posterior probability. Nodes with “-” indicate no bootstrap support. Yellow square represents shrubs, yellow circles represents herbs, and yellow ovals represents semi-shrubs

Maximum likelihood and Bayesian statistical inference methods yielded equivalent topologies for the plastome and ETS. Neither the plastid tree nor the ETS could recover the monophyly of *Ajania* and *Phaeostigma* (Fig. 2, Fig. S1A-I), both of which are highly supported by the internal clades of phylogeny. However, it is clear that the phylogeny of the plastid genome has much higher support across clades (Fig. 2). *Stilpnolepis centiflora* is nested within the *Phaeostigma* clade. *Artemisia* and *Chrysanthemum* are sister groups to *Ajania*. *A. pacifica* is clustered with *Chrysanthemum* into a single branch. By comparing the species tree and the ETS tree, we detected nucleoplasmic conflict. *A. ramosa* is sister to *Artemisia* in the plastid phylogeny and sister to *Brachanthemum* in the ETS tree. *A. variifolia* is sister to *Stilpnolepis*, while in the ETS phylogeny, it clusters with *Ajania* species.

We observed that all three of these species that did not cluster with the main lineages of *Ajania* showed some morphological differences. The species of the *Phaeostigma* lineage (*A. variifolia* + *A. ramosa*) are both shrubs (yellow squares in Fig. 2). *A. pacifica* has marginal ligulate florets, which is clustered with *Chrysanthemum* in plastid and ETS phylogeny. The main lineages of *Ajania* (*A. khartensis* + *A. przewalskii* + *A. tenuifolia* + *A. fruticulosa* + *A. nematoloba*) show a tendency to evolve from herbs (yellow circles) to semi-shrubs (yellow ovals).

### Analysis of selection pressure on the plastid gene

We calculated the selection pressure for 68 plastid genes. The mean dN, dS and dN/dS for all genes ranged from 0.0001 ~ 0.0991, 0.0001 ~ 0.3170 and 0.0001 ~ 0.9526, respectively (Fig. S5). Most genes had dN/dS values less than 0.5, indicating that these genes were mainly subject to purifying selection. The *psbH* and *ycf2* genes had higher dN/dS (> 0.5), indicating that both genes may have undergone positive selection.

### Gene trees landscape

**Fig. 3:**
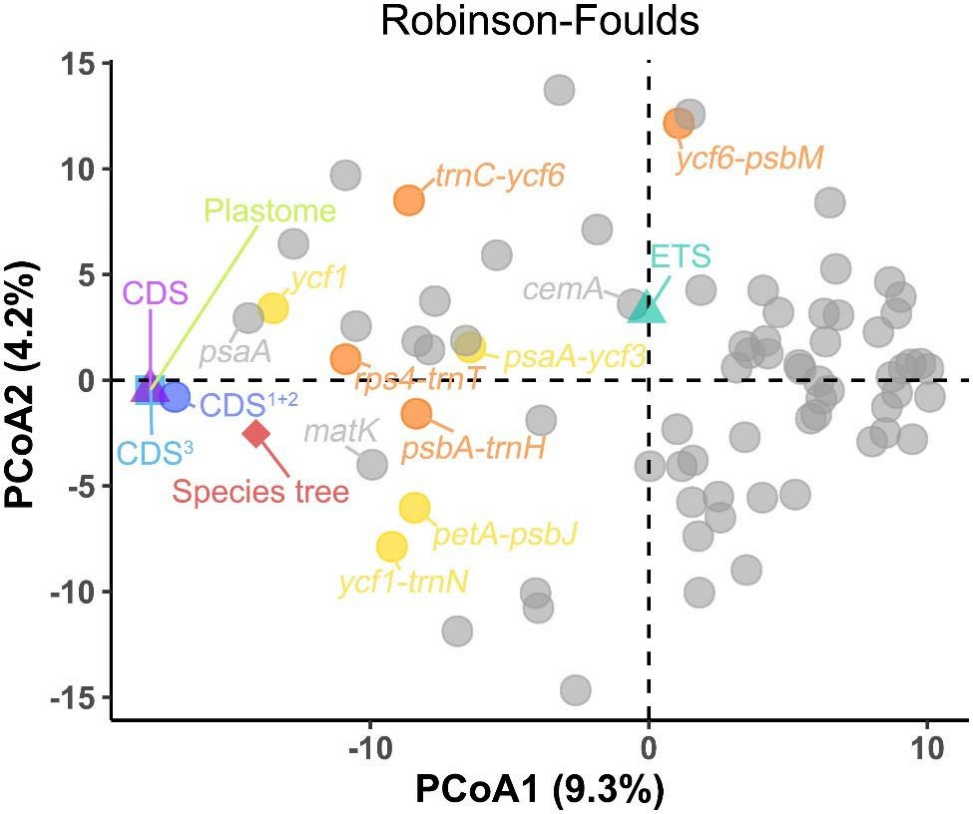
Discordance of phylogeny based on plastid and ETS sequences. Principal coordinate analysis depicting ordinations of ML tree (colour), 68 plastid protein-coding gene (PCG) trees (grey), the DNA markers tree (yellow), and the tree of highly polymorphic region sequences (orange) using unrooted Robinson-Foulds algorithms

We used PCoA to explore the inconsistency of the gene trees. The results showed that the phylogenetic results based on whole plastome and CDS inferences are highly consistent, while there are differences with the species trees (Fig. 3). Individual gene trees showed greater variation. The first and second axes of PCoA explained 9.4% and 4.1% of the variation in tree topology, respectively. The gene trees for *ycf1* and *psaA* (Fig. S1M-N) were closer to the species tree than to the other genes. The *cemA* gene tree (Fig. S1J) was closer to the ETS tree, but they provided limited support for phylogeny.

### Comparative analysis of the structural features of the plastomes

**Fig. 4:**
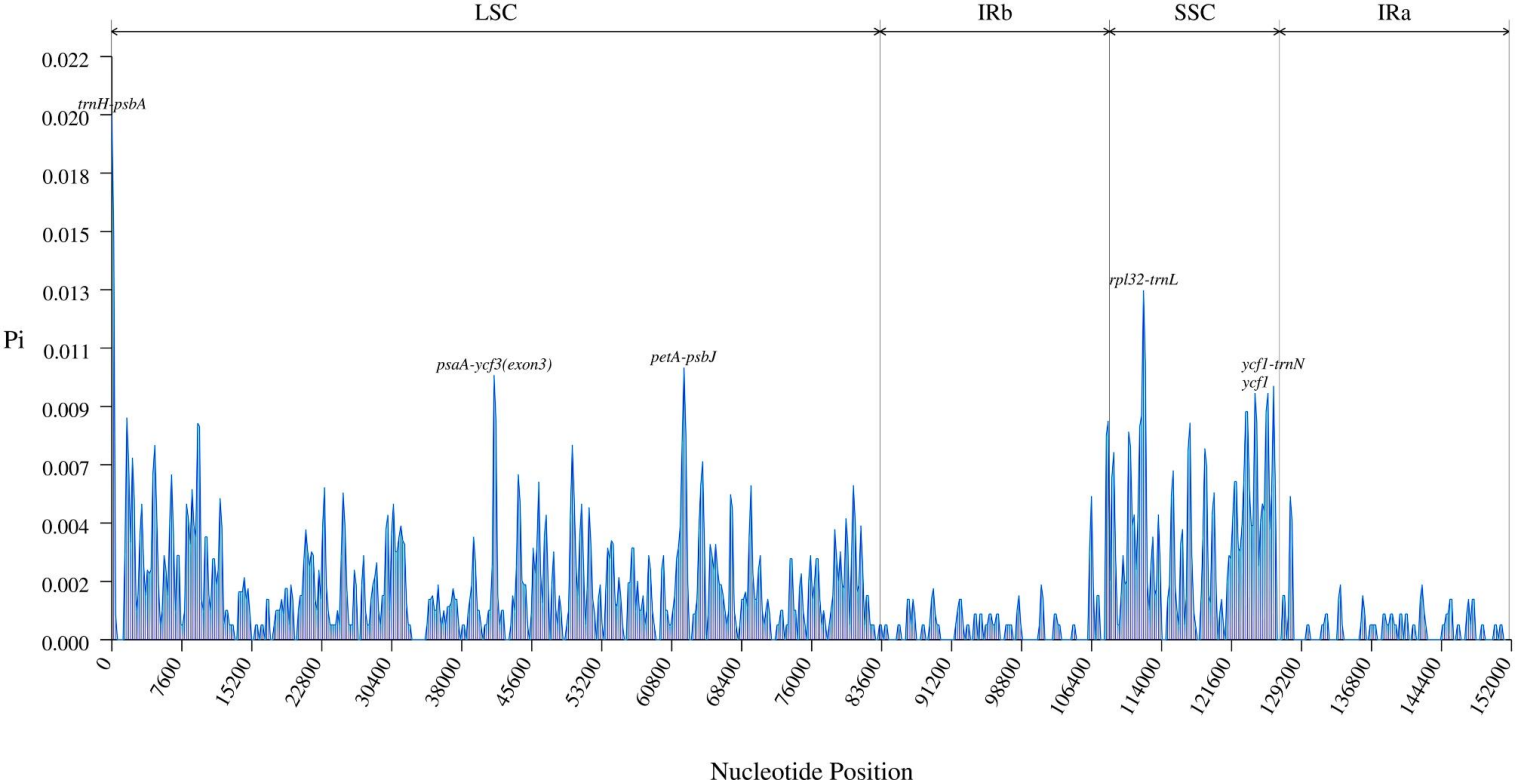
Sliding-window analysis of the whole plastomes for eight *Ajania* species. The X-axis denotes the midpoint position of a window. Y-axis shows nucleotide diversity (Pi) of each window

The results of nucleotide diversity (Fig. 4) and mVISTA analysis (Fig. S2) of the *Ajania*s’ plastomes showed that the plastome sequences of the genus were conserved. Genes located in the IR region are more conserved than those in other regions. We detected six highly polymorphic regions based on Pi values (> 0.009), including *trnH*-*psbA*, *psaA*-*ycf3*, *petA*-*psbJ*, *rpl32*-*trnL*, *ycf1*, and *ycf1*-*trnN*.

**Fig. 5:**
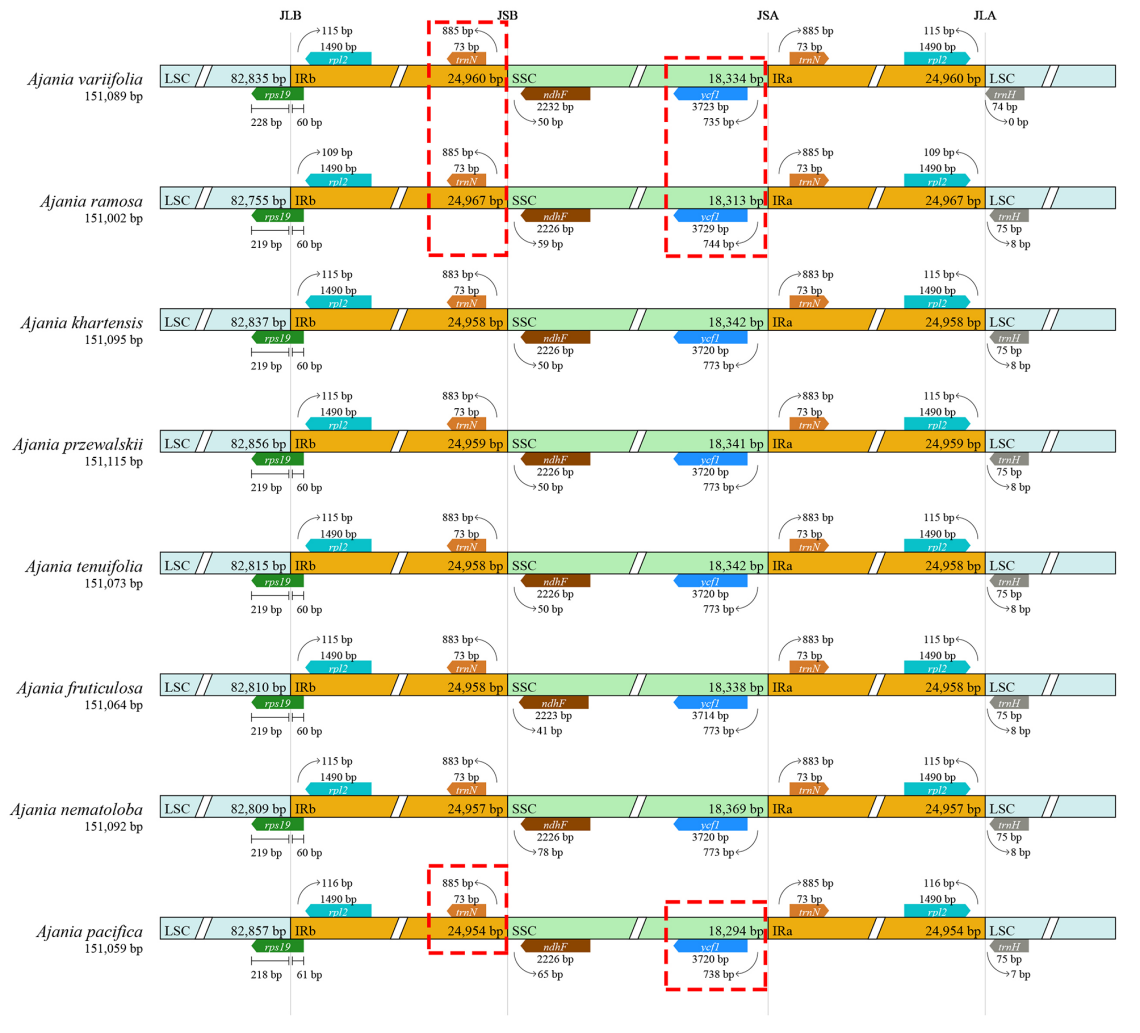
Comparison of the single copy-inverted repeat junctions among the eight *Ajania* species. JLB, JSB, JSA and JLA: LSC/IRb, SSC/IRb, SSC/IRa and LSC/IRa, respectively. The red dashed box shows the main variants in the SSR/IR region of the plastomes of *Ajania* species

We compared the boundaries of IRs and SCs of eight *Ajania* species and found them to be conserved (Fig. 5). The boundary between LSC and IRb occurs in *rps19*, the boundary between IRb and SSC is within *trnN-ndhF* spacer, the boundary between SSC and IRa is in the *ycf1*-*trnN* spacer, and the boundary between IRa and LSC is in the *rpl2*-*trnH* spacer. Combined with the phylogenetic results, *A. variifolia*, *A. ramosa* and *A. pacifica* were observed a tendency for the *ycf1* gene to expand further toward the boundary region. In contrast, the *trnN* genes of these three species have a tendency to contract further within the IRb region (as shown in the red dashed box in Fig. 5).

**Fig. 6:**
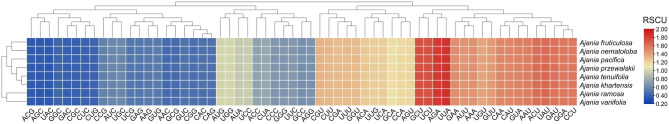
The RSCU values of all merged CDSs among eight *Ajania* plastomes. Color key: the red values indicated higher RSCU values while the blue values indicated lower RSCU values

There were 29 synonymous codons with RSCU values greater than 1, with the UUA codon encoding leucine having the highest RSCU value (1.87 ~ 1.88), followed by the AGA codon encoding arginine (RSCU = 1.83 ~ 1.84), and the AGC codon encoding serine having the lowest RSCU value (0.36 ~ 0.37). The relative synonymous codon usage preferences of the eight *Ajania* plastomes were generally consistent (Fig. 6), with minor differences. The results of species clustering based on codon preference were generally consistent with the phylogenetic results, except for *A. pacifica*, which showed differences.

### Plastid genomic repeat sequence

In the eight *Ajania* species, we detected three long dispersed repeats (LDRs) patterns (Table S5): forward repeats, reverse repeats and palindrome repeats. The results showed that there were small differences in the distribution of repeat sequences in the plastomes of different species (Fig. S3). The reverse repeats were only present in the LSC region of *A. nematoloba*, *A. ramosa* and *A. tenuifolia*, but were located in the spacer region (IGS) of the LSC and intron regions in *A. ramosa* and *A. tenuifolia.* Both contain a reverse repeat in the *atpA*-*trnR* and *clpP* intron regions, while the reverse repeats of *A. nematoloba* are mainly located in the intron region of the *rps16* gene in the LSC region.

The results of the simple sequence repeats (SSRs) analysis showed that we detected a total of seven simple repeat patterns in *Ajania*’s plastomes, with the highest number of single-nucleotide repeats (Table S6). The distribution patterns of single and dinucleotide repeats in the IR region were largely consistent. Complex repeats and pentanucleotide repeats showed differential distributions in the plastid genomes (Fig. S4). *A. nematoloba* lacked pentanucleotide repeats and was missing trinucleotide repeats in the LSC intron region. *A. variifolia*, *A. ramosa* and *A. pacifica* lacked the complex repeats for the SSC region.

## Discussion

### Phylogeny of the genus *Ajania*

Previous morphological and molecular phylogenetic studies have clarified *Ajania*’s phylogenetic position in relation to closely related to *Chrysanthemum*, *Brachanthemum*, *Leucanthemella*, *Kaschgaria*, and *Artemisia* [10, 12, 14, 19]. However, the findings of these studies show a nested phylogenetic relationship between *Ajania* and *Chrysanthemum*. The precise internal relationship of *Ajania* remains uncertain, and the classification of the genus is not well resolved. Utilizing the plastid dataset and ETS sequences, we validated the taxonomic treatment and found new insights on *Ajania*’s major relationships.

Our results reveal that *Phaeostigma* is distantly related to *Ajania* and suggest the feasibility of separating *Phaeostigma* from *Ajania* [14, 15]. *Phaeostigma* lineage, including *A. ramosa* (= *P. ramosum*) and *(A) variifoliav* (= *P. variifolium*), were shown to be more closely linked to *Artemisia* than to the major *Ajania* lineages (*A. khartensis* + *A. przewalskii* + *A. tenuifolia* + *A. fruticulosa* + *A. nematoloba*) in the plastid phylogeny (Fig. 2 and Fig. S1). The phylogenetic position of *P. ramosum* and *P. variifolium* further supports the notion that their comparable capitulum characteristics and geographical distribution are the product of convergent evolution in similar settings [13, 16]. In this study, there were no significant affinities between *P. ramosum* and *P. variifolium*, which is consistent with previous studies [11, 14]. We reveal the nucleoplasmic conflict in the plastid and ETS phylogenies of *P. variifolium*: in the plastid phylogeny it shows affinities with *(B) pulvinatum* and *S. centiflora*, while in the ETS phylogeny it is clustered with the major *Ajania* lineages. Previous molecular phylogenetic analyses utilizing plastid sequences have provided robust evidence for the inclusion of *P. ramosum* and *P. variifolium* within the taxonomic classification of *Phaeostigma* [13, 14]. However, metabolomics-based phylogeny revealed that *P. ramosum* and *P. variifolium* are located in different lineages, and both have nested phylogenetic relationships with *Ajania* and *Chrysanthemum* [11]. Regarding the shifting locations of *P. ramosum* and *P. variifolium* within phylogenetic analyses utilizing various datasets, it is postulated that this phenomenon might potentially be attributed to chloroplast capture, introgression, or adaptive expression. Comprehensive investigation is required to ascertain the precise factors contributing to these changes.

Both *Phaeostigma* and *Ajania* are polyphyletic in this study, with *Artemisia* and *Chrysanthemum* being sister groups of both, as previously documented [13, 14]. *Phaeostigma* and *Ajania* are considered transitional taxa between *Artemisia* and *Chrysanthemum* due to their strong affinity [26]. Our plastid phylogeny demonstrates that *Phaeostigma* diverged earlier than *Artemisia* (Fig. 2 and Fig. S1A-H), whereas *Ajania* forms a main lineage with *Chrysanthemum*. The strong affinity between the genera *Ajania* and *Chrysanthemum* makes it difficult to distinguish the two phylogenetically, and their connection, as well as patterns of diversification and development, remain extensively debated. Earlier research has suggested that *Ajania* be included in *Chrysanthemum* [8, 10, 12]. Unfortunately, not all investigations have supported this theory [11]. There are several unresolved concerns about the taxonomic classification of *Ajania* and *Chrysanthemum*. Given the small number of species included in this study, we remain cautious about combining the genera *Ajania* and *Chrysanthemum*.

A discernible pattern of evolutionary progression from herbaceous to semi-shrub forms was observed within the main *Ajania* lineages (Fig. 2). In contrast to the habitats of other species, *A. khartensis*, *A. przewalskii*, and *A. tenuifolia* exhibit a preference for habitats characterized by favorable water and heat conditions, such as hillside grasslands. On the other hand, *A. fruticulosa* and *A. nematoloba* prefer desert and semi-desert environments. The reduction in *Ajania* leaf abundance can be attributed to the alterations in its habitat, potentially indicating its capacity to adapt to arid environments [27, 28]. The observed evolutionary inclination could potentially be associated with the expansion and differentiation of *Ajania* in the East Asian region. The *Ajania* lineage experienced either in situ diversification or colonization. This diversification was influenced by the geological processes of mountain-building on the Qinghai-Tibetan Plateau, as well as the climatic fluctuations in East Asia [13]. Nonetheless, the specific evolutionary trajectory within *Ajania* remains uncertain, and a more comprehensive sampling is necessary to conduct more thorough analysis. Moreover, all five species’ plastid genomes include complex repeats in the SSC region (Fig. S4), although they are dispersed differently. These repeat sequences might be possibilities for species molecular calibration.

The affinities of *Phaeostigma* and *Stilpnolepis*, both of which have discoid capitula, were described for the first time in this study. *Stilpnolepis* predominantly inhabits arid desert regions [29], while *Phaeostigma* is primarily distributed in the Qinghai-Tibetan Plateau and its surrounding regions [14]. The phylogenetic location of *Phaeostigma* and *Stilpnolepis* indicates cyto-nuclear discordance (Fig. 2). In conjunction with nuclear gene-based phylogenetic investigations [10, 13, 14], we hypothesize that the two may have experienced chloroplast capture events or secondary interactions early in species formation. Subsequently, they underwent convergent evolution in similar habitats in different regions, resulting in highly similar capitula characteristics.

*A. pacifica* is mainly distributed in Japan and usually clustered with cultivated *Chrysanthemum* species [30]. There is incomplete reproductive isolation between the two [5]. During our examination of specimens and plants, we found that some of the *A. pacifica* marginal florets had incomplete laminae. This confusion has been suggested in previous molecular phylogenetic studies as a possible result of secondary contact, with gene infiltration leading to incomplete morphological differentiation [13, 31, 32]. Further study is needed to determine the taxonomic status of this species and its relationships with the genus *Chrysanthemum*.

The observed topological inconsistencies between the concatenated and ASTRAL topologies could be attributed to incomplete genealogical sorting (ILS) [33], or to the general limitations of ASTRAL, as many or most plastid genes contain motifs that are largely devoid of phylogenetic information. Studies have shown ASTRAL to be more accurate under high ILS conditions [34]. While the extent of ILS in the present dataset is unknown, major clades of Asteraceae have experienced rapid radiation [35, 36], a condition often associated with high ILS.

Our results reveal a nucleoplasmic conflict between *Ajania* and its relatives, which may have a complicated evolutionary history, including involved rapid diversification (hybridization, ILS, polyploidy, etc.) and gene infiltration (including chloroplast capture) [33, 37, 38]. In addition, convergent evolution, gene duplication, evolutionary rate heterogeneity and long branch attraction also have important effects on these inconsistencies [39]. Hybridization may be the primary source of nucleoplasmic conflicts for species on distinct evolutionary branches from plastid and nuclear phylogeny [40]. It is not rare for *Ajania* and its cousins to hybridize [5, 41, 42]. Although these crossings contributed significantly to germplasm innovation and genetic enhancement of horticulture plants, they also enhanced the phylogenetic complexity of *Ajania* and its relatives.

### Structural features of the plastid genome of *Ajania*

For the first time, we compared the plastid genomes of *Ajania* species from distinct clades. We discovered that, like other angiosperms [43], *Ajania*s’ plastomes had a highly conserved structure, gene content, and gene order. IR contraction and expansion frequently result in plastome length variations [44]. The *Ajania*s’ plastomes exhibit expansion and contraction corresponding to the phylogenetic position of the different clades (Fig. 5). This implies that in *Ajania*, plastome characteristics may reflect partial species phylogenetic relationships. Additionally, codon preferences exhibit a similar pattern (Fig. 6). Nevertheless, the study’s sampling breadth was restricted, and more research is needed to verify whether both accurately reflect the evolutionary connections of all *Ajania* species.

The advancement of molecular markers has greatly aided in species identification and systematic categorization. Currently, the *rpl16* gene intron region, *trnL-F* and intergenic spacer regions (*psbA*-*trnH*, *trnC*-*ycf6*, *ycf6*-*psbM*, *trnY*-*rpoB* and *rpS4*-*trnT*) have been used for DNA markers and phylogenetic inference in Asteraceae [12, 14]. Except of *psbA*-*trnH*, these sequences show limited nucleotide diversity (Fig. 4) and are therefore only of limited utility for phylogenetic categorization. This may have contributed to early phylogenetic studies’ ambiguity about interspecific connections within *Ajania*. As a result, developing high-resolution and polymorphic molecular markers for the genus *Ajania* is critical. The highly polymorphic regions found in this study (*psaA*-*ycf3*, *petA*-*psbJ*, *rpl32*-*trnL*, *ycf1*, *ycf1*-*trnN*) may serve as a model for the creation of molecular markers. In addition, the *ycf1* gene is well recovered from the major lineages of *Ajania* (Fig. 3 and Fig. S1N). Compared to the *psaA* gene, the *ycf1* gene provides more phylogenetic variation and higher support (Fig. S1M-N) as a candidate for molecular markers with species identification implications [45, 46].

Repeating sequences in the plastome represent a possible mutational hotspot [47]. Slip chain mismatch and faulty recombination will lead to genomic sequence variation and rearrangements that are critical in species evolution [48, 49]. Previous studies demonstrated that repeated sequences may be employed for plant population genetics and the identification of polymorphic loci [50, 51]. In this study, the *Ajania* plastomes were conserved, and LDRs were distributed in a generally consistent pattern. Forward and palindrome repetitions were abundant in the plastomes, while reverse repeats were distributed differently (Fig. S3AB). The IGS area included the greatest number of SSRs in this research, which were also detected in the majority of plants [50]. Mononucleotide repeats and tetranucleotide repeats were widespread in the plastome, whereas dinucleotide repeats, trinucleotide repeats, pentanucleotide repeats and complex repeats had a preferential distribution in the plastome (Fig. S4). This may correspond to the high variability of the IGS region. Differences in the distribution of repetitive sequences in the plastid genome may provide molecular markers for species identification [52].

The utilization of nucleotide substitution rate as a significant molecular marker for gene evolution and natural selection has been extensively employed [53]. A ratio of dN/dS larger than 0.5 is considered to be an appropriate threshold for the identification of candidate genes in the context of adaptive evolution [54]. In this study, *psbH* and *ycf2* were identified as having accelerated substitution rates in *Ajania* (Fig. S5). The *psbH* gene, which has been associated with the oxygen-evolving core complex [55], is ubiquitously present in the majority of plant species. The presence of this component within the photosystem II reaction center complex is essential for the processes of photoinhibition repair and efficient assembly [56]. The ycf2 gene plays a crucial role in the transmembrane transport of ATP [57]. Additionally, it has been identified as the biggest known plastid gene in angiosperms [58]. This gene also has a strong phylogenetic signal, with high family-tribe level polymorphism [59], and it can provide solid evidence for phylogenetic connections across angiosperm populations instead of using a multigene strategy [60]. Plastid genes in *Ajania* may experience selective pressure, potentially influencing processes such as photosynthesis and ATP transfer. Differences in the rates of nucleotide substitution among specific genes could potentially be attributed to variations in the overall mutation rates across the genome.

## Conclusions

The first results of employing a phylogenetic dataset to examine the phylogeny of *Ajania* are presented here. Our findings validated the early taxonomy reclassification, and showed a nucleoplasmic conflict between *Ajania* and its relatives. The similarities in capitulum characteristics between *Phaeostigma* and *Ajania* are most likely the consequence of convergent evolution. Comparative genomic studies found significant evolutionary rate heterogeneity, genetic variation between plastid genes, and plastid gene phylogenetic heterogeneity. In certain species, plastome structural traits may reveal evolutionary connections. We propose six potential molecular marker sequences for species identification and speculate that the *ycf1* gene may better depict *Ajania*’s evolutionary connections than other genes. Our results enhance the understanding of the phylogenetic relationships of *Ajania*. We hope that this study can contribute to further analysis of *Ajania* for other researchers.

## Materials and methods

### Taxon sampling, DNA extraction, and sequencing

We collected a total of six species of *Ajania* in the field, all from Qinghai Province in China. Before collecting the samples, we got oral permission from the local government after applying with introduction letters of Northwest Institute of Plateau Biology, Chinese Academy of Sciences. Voucher specimens of six *Ajania* species were identified by Faqi Zhang, and were deposited into the Qinghai-Tibetan Plateau Museum of Biology (HNWP), Northwest Institute of Plateau Biology, Chinese Academy of Sciences (voucher ID numbers: Art02n for *A. khartensis*; Art03 for *A. nematoloba*; Art04 for *A przewalskii*; Art05 for *A ramosa*; Art07 for *A. tenuifolia*; QXA0018 for *A. fruticulosa*). The detailed information was shown in Additional file 1: Table S1.

Fresh leaves were dried on silica gel and stored at -20 °C. Total DNA was extracted from frozen leaf tissue using a modified CTAB method [61]. The genomic DNA library was generated using NEB Next® UltraTM DNA Library Prep Kit for Illumina (NEB, United States) following the manufacturer’s recommendations, and index codes were added to each sample and sequenced on an Illumina HiSeq 2500 sequencer (San Diego, CA, United States) using the paired-end option (2 × 150 bp). The quality of raw reads was evaluated by FastQC v0.11.8 (https://www.bioinformatics.babraham.ac.uk/projects/fastqc/). Low-quality reads were filtered and trimmed by Trimmomatic v0.33 [62].

### Assembly and annotation of the plastid genome and ETS

For the plastome, we used GetOrganelle v1.7.5 [63] for assemble the plastome. The assembled plastomes were annotated using PGA [64] and CPGAVAS2 [65]. The start/stop codons and intron/exon boundaries of the plastomes were manually checked and adjusted. The sequences were submitted to ORGDRAW’s online tool for chloroplast genome visualization [66].

For ETS, we first constructed a reference sequence pool using the eight published ETS sequences of Asteraceae from GenBank (Table S2); then combined with our previous sequencing data, we performed de novo assembly using easy353 [67]; and finally checked and trimmed using BLAST v2.13.0+ [68].

### Phylogenetic analysis

For the plastome, we used PhyloSuite v1.2.2 [69] for protein-coding sequences (CDS) extraction in conjunction with the published GenBank plastid genomes of *Ajania* and its relatives (Table S1). MAFFT v7.310 [70] was used for sequence comparisons, and the parameters were set to G-INS-I (accurate). CDS sequences (*atpH*, *petL*, *psbK*, *psbL*, *psbJ*, *psbM*, *psbN*, *psbT*, *rpl2*, *rpl16*, and *rpl23*) with differences of less than 4 bp were manually removed. The matched datasets were cut using GBlock [71] to remove poorly matched regions and divergent regions. Six datasets were constructed: dataset I with 68 CDSs concatenated; dataset II with 68 CDS first and second codons concatenated (CDS^1 + 2^); dataset III with 68 CDS third codons concatenated (CDS^3^); dataset IV with complete plastomes; dataset V with ETS sequences; dataset VI with 68 CDS in parallel.

For datasets I-V, phylogenetic analyses were performed using maximum likelihood (ML) and Bayesian (BI) methods, with *Aster tataricus* and *Rhodanthemum hosmariense* [10] respectively serving as outgroups for plastome and ETS phylogenetic analyses. These outgroups were selected due to their distant phylogenetic relationship with *Ajania* and its related taxa. For ML analysis, ModelFinder [72] inferred the best partitioning scheme and optimal evolutionary model based on the Bayesian Information Criterion (BIC) (Table S3). The ML tree was then constructed using IQtree v2.0.3 [73] with fast natural replicates (rapid bootstrap replicates) set to 1000. For Bayesian analyses, ModelFinder inferred the best partitioning scheme and the best evolutionary model based on the Corrected Akaike Information Criterion (AICc), followed by the construction of BI trees using Mrbayes [74]. Each Bayesian analysis was performed through two independent runs of four 1,000,000 generations Monte Carlo Markov chains (MCMC), sampled every 1000 generations. After the first 25% of the preheat trees (burn-in = 25%) were burned, the remaining trees generated consistent trees and Bayesian posterior probabilities (PP) were calculated.

For dataset VI, gene trees were constructed using IQtree for each CDS, with rapid bootstrap replicates set to 1000. All gene trees were combined in ASTRAL v.5.7.8 [75] to form a species tree with coalescence. The trees were visualized and edited using Interactive Tree of Life (iTOL) [76].

### Nucleotide substitution rates and landscape tree analysis

To estimate the nucleotide substitution rate, synonymous (dS) and non-synonymous (dN) substitution rates and the ratio of the two, dN/dS, were calculated in paml v4.9 [77] using the codeml option, with codon frequencies using the F3 × 4 model and parameters set to CodonFreq = 2, model = 0 and cleandata = 1.

We mapped the statistical distribution of trees using the Robinson-Foulds algorithm [78] to explore variation in gene trees. ML trees based on CDS^1 + 2^, CDS^3^, the whole plastid genome, CDS and ETS constructs, and species trees were used as datasets. Distances between unrooted trees were calculated using the R package TREESPACE v.1.0.0 [79], with reference to the workflow of Goncalves et al. [80], and the first two principal coordinate analysis (PCoAs) were estimated. Results were visualised using ggplot2.

**Genomic structure and comparative analysis of** ***Ajania*s’ plastomes**.

For the plastomes of the eight *Ajania* species included in this study, DNAsp6 [81] was used to calculate nucleotide diversity (Pi) with a window length set to 400 bp and a step size set to 200 bp. ML trees were constructed for the detected highly polymorphic regions and DNA markers (*psbA*-*trnN*, *trnC*-*ycf6*, *ycf6*-*psbM*, *rps4*-*trnT*) used in previous molecular phylogenetic studies [12, 14]. The trees were compared and visualized using TREESPACE v.1.0.0 [79]. Whole plastome similarity analysis and visualization was performed using the mVISTA online platform [82] to implement and Shuffle-LAGAN [83] comparison mode was selected. CPJSdraw (http://112.86.217.82:9919/#/home) was used to visualize the gene distribution at the junctions of the IR/SC regions of plastid genome. codonW v1.3 (https://codonw.sourceforge.net/) is used for the detection of relative synonymous codon usage (RSCU) for all plastid genes.

REPuter [84] was used to detect LDRs larger than 10 bp with > 90% sequence similarity in the plastome, with the maximum and minimum repeat length set to 50 bp and 30 bp, respectively, and the Hamming distance set to 3. Web-MISA [85] was used to identify SSRs with the following parameters: ten repetitions for mononucleotide motifs, five for dinucleotide motifs, four for trinucleotide motifs and three for tetranucleotide, pentanucleotide and hexanucleotide motifs. The R package ggplot2 was used to visualization.

### Electronic supplementary material

Below is the link to the electronic supplementary material.

Supplementary Material 1

## Data Availability

Six plastome and nine ETS sequence data generated in this study are available in GenBank of the National Center for Biotechnology Information (NCBI) Names of the repository/repositories and accession number(s) can be found in the Additional File (Table S2). The datasets generated and/or analysed during the current study are available in the GenBank repository, https://www.ncbi.nlm.nih.gov/genbank/.
