# Supplementary material for: Structural divergence and phylogenetic relationships of Ajania (Asteraceae) from plastomes and ETS
Source: BMC Genomics. 2023 Oct 10;24:602. doi: 10.1186/s12864-023-09716-4 (PMC10566131; doi:10.1186/s12864-023-09716-4)
Supplement: Supplementary file 1 — Supplementary Material 1 [file 12864_2023_9716_MOESM1_ESM.docx]

**Structural divergence and phylogenetic relationships of *Ajania* (Asteraceae) from plastomes and ETS**

**Additional**

**Jingya Yu ^1, 2,^ #, Yun Han ^1, 2, #^, Hao Xu ^1, 2^, Shuang Han ^1, 2^, Xiaoping Li ^1, 2^, Yu Niu ^1, 2^, Shilong Chen ^1^, Faqi Zhang ^1, 3*^**

^1^ Key Laboratory of Adaptation and Evolution of Plateau Biota, Northwest Institute of Plateau Biology & Institute of Sanjiangyuan National Park, Chinese Academy of Sciences, Xining 810008, China

^2^ University of Chinese Academy of Sciences, Beijing 100039, China

^3^ Qinghai Provincial Key Laboratory of Crop Molecular Breeding, Xining 810008, China

^*^ Correspondence: [fqzhang@nwipb.cas.cn](mailto:fqzhang@nwipb.cas.cn)

# Legends

**Table S1.** Collection and sequencing information of six *Ajania* species.

**Table S2.** Characteristics of plastomes and ETS sequences.

**Table S3.** The best nucleotide substitution model as found by ModelFinder for various datasets using the Corrected Akaike Information Criterion (AICc) and Bayesian Information Criterion (BIC).

**Table S4.** Gene content of plastomes of *Ajania*.

**Table S5.** Sample distribution of long dispersed repeats (LDRs) in the plastome for eight *Ajania* species. F indicates forward repeats, P indicates palindromic repeats, and R indicates reverse repeats.

**Table S6.** Sample distribution of simple repeat sequence (SSR) in the plastome for eight *Ajania* species. p1 indicates single nucleotide repeats, p2 indicates dinucleotide repeats, p3 indicates trinucleotide repeats, p4 indicates tetranucleotide repeats, p5 indicates pentanucleotide repeats, p6 indicates hexanucleotide repeats, and c indicates complex repeats.

**Fig. S1.** Maximum likelihood trees, Bayesian trees and gene trees constructed based on different data sets. The numbers on the branches are the maximum likelihood tree support and Bayesian posterior probabilities; no numbers indicate that the branch has no support or posterior probability.

**Fig. S2.** mVISTA-based sequence identity plot of eight *Ajania* plastomes compared using *A. fruticulosa* as a reference. Blue represents coding regions, pink represents non-coding regions and gray arrows point at gene

**Fig. S3.** Sample distribution of long dispersed repeats (LDRs) in the plastome for eight *Ajania* species: A represents the distribution pattern of LDRs in the large single copy (LSC), the small single copy (SSC), and the inverted repeat (IR); B represents the distribution pattern of LDRs in exon, the spacer region (IGS), and intron regions. F indicates forward repeats, P indicates palindromic repeats, and R indicates reverse repeats.

**Fig. S4.** Sample distribution of simple repeat sequence (SSR) in the plastome for eight *Ajania* species: A represents the distribution pattern of SSRs in the large single copy (LSC), the small single copy (SSC), and the inverted repeat (IR); B represents the distribution pattern of SSRs in exon, the spacer region (IGS), and intron regions. p1 indicates single nucleotide repeats, p2 indicates dinucleotide repeats, p3 indicates trinucleotide repeats, p4 indicates tetranucleotide repeats, p5 indicates pentanucleotide repeats, p6 indicates hexanucleotide repeats, and c indicates complex repeats.

**Fig. S5.** The estimations of nonsynonymous (dN), synonymous (dS) substitution rates and dN/dS of plastid protein-coding genes (CDS). The grey line denotes a dN/dS threshold of 0.5 to screen positive selection of genes.

**Table S1.** Collection and sequencing information of six *Ajania* species.

| Species | Voucher ID | Raw Data(GB) | Clean Data(GB) | Q30 | GC content | Latitude | Longitude |
| --- | --- | --- | --- | --- | --- | --- | --- |
| *Ajania khartensis* (Dunn) C.Shih | Art02 | 15.8 | 15.7 | 94.2 | 36.6 | 38.443 | 99.565 |
| *Ajania nematoloba* (Hand.-Mazz.) Y.Ling & C.Shih | Art03 | 16.1 | 16.0 | 93.8 | 36.4 | 35.804 | 102.691 |
| *Ajania przewalskii* Poljakov | Art04 | 13.7 | 13.6 | 93.7 | 36.5 | 35.339 | 99.913 |
| *Ajania ramosa* (C.C.Chang) C.Shih | Art05 | 14.6 | 14.5 | 93.8 | 35.6 | 35.697 | 100.261 |
| *Ajania tenuifolia* Tzvelev | Art07 | 13.7 | 13.6 | 94.4 | 36.5 | 35.348 | 99.935 |
| *Ajania fruticulosa* (Ledeb.) Poljak. | QXA0018 | 6.8 | 6.8 | 89.5 | 36.5 | 37.311 | 96.637 |

**Table S2.** Characteristics of plastomes and ETS sequences.

| Organism | Voucher ID | Accession number (plastome) | Plastid length (bp) | LSC length (bp) | SSC length (bp) | IRs length (bp) | Gene | tRNA | rRNA | Pseudogenes | Accession number (ETS) | ETS length (bp) |
| --- | --- | --- | --- | --- | --- | --- | --- | --- | --- | --- | --- | --- |
| *Ajania fruticulosa* | QXA0018 | OQ680030 | 151064 | 82810 | 18338 | 24958 | 87 | 37 | 8 | *Ψycf1* | OQ703616 | 1213 |
| *Ajania khartensis* | Art02 | OP723181 | 151095 | 82,837 | 18,342 | 24,958 | 87 | 37 | 8 | *Ψycf1* | OQ703610 | 1211 |
| *Ajania nematoloba* | Art03 | OP723182 | 151092 | 82,809 | 18,369 | 24,957 | 87 | 37 | 8 | *Ψycf1* | OQ703611 | 1214 |
| *Ajania pacifica* | - | NC_050690.1 | 151059 | 82857 | 18,294 | 24954 | 87 | 37 | 8 | *Ψycf1* | AB359856 | 1087 |
| *Ajania przewalskii* | Art04 | OP723183 | 151115 | 82,856 | 18,341 | 24,959 | 87 | 37 | 8 | *Ψycf1* | OQ703612 | 1269 |
| *Ajania ramosa* | Art05 | OP723184 | 151002 | 82,755 | 18,313 | 24,967 | 87 | 37 | 8 | *Ψycf1* | OQ703613 | 1980 |
| *Ajania tenuifolia* | Art07 | OP723186 | 151073 | 82,815 | 18,342 | 24,958 | 87 | 37 | 8 | *Ψycf1* | OQ703614 | 1212 |
| *Ajania variifolia* | - | NC_057440 | 151089 | 82,835 | 18,334 | 24,960 | 87 | 37 | 8 | *Ψycf1* | AB359873 | 1080 |
| *Artemisia tangutica* | zhang2018015 | MT701043.1 | 151140 | 82,885 | 1,836 | 24,959 | 87 | 37 | 8 | *Ψycf1* | OQ719615 | 840 |
| *Artemisia vulgaris* | Art42 | OP723270 | 151134 | 82,871 | 18,342 | 24,960 | 87 | 37 | 8 | *Ψycf1* | OQ703615 | 2071 |
| *Aster tataricus* | - | NC_042913 | 152992 | 82,857 | 18,294 | 25,022 | 87 | 37 | 8 | - | - | - |
| *Brachanthemum pulvinatum* | QXA0162 | OP723274 | 151289 | 82,998 | 18,371 | 24,960 | 87 | 37 | 8 | *Ψycf1* | OQ703617 | 2133 |
| *Chrysanthemum boreale* | - | MN909052 | 151098 | 82,878 | 18,312 | 24,954 | 87 | 37 | 8 | *Ψycf1* | AB359814 | 1088 |
| *Chrysanthemum indicum* | - | MH165290 | 151095 | 82,866 | 18,323 | 24,953 | 87 | 37 | 8 | *Ψycf1* | AB359822 | 1086 |
| *Chrysanthemum zawadskii* | - | MW539687.1 | 151137 | 82,881 | 18,350 | 24,953 | 87 | 37 | 8 | *Ψycf1* | AB359846 | 1086 |
| *Leucanthemella linearis* | - | MN883842 | 151395 | 83,080 | 18,391 | 24,962 | 87 | 37 | 8 | - | AB359888 | 1284 |
| *Rhodanthemum hosmariense* |  | - | - | - | - | - | - | - | - | *-* | AB359891 | 1080 |
| *Stilpnolepis centiflora* | - | NC_052884 | 151017 | 82,782 | 18,395 | 24920 | 87 | 37 | 8 | - | AB359877 | 1265 |

**Table S3.** The best nucleotide substitution model as found by ModelFinder for various datasets using the Corrected Akaike Information Criterion (AICc) and Bayesian Information Criterion (BIC).

| Patition region | Model (AICc) | | | | | Model(BIC) | | | | |
| --- | --- | --- | --- | --- | --- | --- | --- | --- | --- | --- |
|  | Dataset I | Dataset II | Dataset III | Complete platomes | ETS | CDS | CDS12 | CDS3 | Complete platomes | ETS |
| other | - | - | - | GTR+F+I | HKY+F+I | - | - | - | K3Pu+F+I | GTR+F+I |
| *accD* | GTR+F+I | TPM3u+F+I | GTR+F | - | - | TPM3u+F+I | GTR+F+I | TVM+F+G4 | - | - |
| *atpA* | GTR+F+G4 | TVM+F+I | GTR+F | - | - | TVM+F+G4 | GTR+F+I | TVM+F+G4 | - | - |
| *atpB* | GTR+F+I | TVM+F+I | GTR+F | - | - | TVM+F+G4 | GTR+F+I | TVM+F+G4 | - | - |
| *atpE* | GTR+F | HKY+F+I | GTR+F | - | - | K3Pu+F+I | F81+F | TVM+F+G4 | - | - |
| *atpF* | GTR+F+G4 | TVM+F+I | GTR+F | - | - | TVM+F+G4 | HKY+F | TVM+F+G4 | - | - |
| *atpH* | GTR+F+I | HKY+F+I | GTR+F | - | - | K3Pu+F+I | GTR+F+I | TVM+F+G4 | - | - |
| *atpI* | GTR+F+I | TVM+F+I | GTR+F+I | - | - | K3Pu+F+I | F81+F | TVM+F+G4 | - | - |
| *ccsA* | GTR+F+I | TPM3u+F+I | GTR+F+G4 | - | - | TVM+F+I | GTR+F+I | GTR+F+G4 | - | - |
| *cemA* | GTR+F+I | K3Pu+F | GTR+F+I | - | - | TVM+F+G4 | GTR+F+I | TVM+F+G4 | - | - |
| *clpP* | GTR+F+G4 | TVM+F+I | GTR+F+G4 | - | - | TVM+F+G4 | GTR+F+I | TVM+F+G4 | - | - |
| *infA* | GTR+F+G4 | TVM+F+I | GTR+F | - | - | TVM+F+G4 | HKY+F+I | GTR+F+G4 | - | - |
| *matK* | GTR+F+G4 | TPM3u+F+I | GTR+F+G4 | - | - | TVM+F+I | GTR+F+I | GTR+F+G4 | - | - |
| *ndhA* | GTR+F+I | K3Pu+F | GTR+F | - | - | TVM+F+G4 | GTR+F | GTR+F+G4 | - | - |
| *ndhB* | GTR+F+I | HKY+F+I | GTR+F | - | - | K3Pu+F+I | GTR+F+I | TPM3u+F | - | - |
| *ndhC* | HKY+F | TVM+F+I | GTR+F | - | - | K3Pu+F+I | F81+F | TVM+F+G4 | - | - |
| *ndhD* | F81+F+I | TPM3u+F+I | GTR+F+G4 | - | - | TVM+F+I | F81+F+I | GTR+F+G4 | - | - |
| *ndhE* | GTR+F+I | TVM+F+I | GTR+F+G4 | - | - | TVM+F+G4 | GTR+F+I | GTR+F+G4 | - | - |
| *ndhF* | GTR+F+I | K3Pu+F | GTR+F | - | - | TVM+F+I | GTR+F | GTR+F+G4 | - | - |
| *ndhG* | GTR+F+I | K3Pu+F | GTR+F+G4 | - | - | TVM+F+G4 | GTR+F | TVM+F+G4 | - | - |
| *ndhH* | GTR+F+I | TVM+F+I | GTR+F+G4 | - | - | TVM+F+G4 | GTR+F+I | GTR+F+G4 | - | - |
| *ndhI* | GTR+F+I | TVM+F+I | GTR+F | - | - | TVM+F+G4 | GTR+F+I | TVM+F+G4 | - | - |
| *ndhJ* | GTR+F+I | TVM+F+I | GTR+F+G4 | - | - | K3Pu+F+I | GTR+F+I | TVM+F+G4 | - | - |
| *ndhK* | GTR+F+I | TVM+F+I | GTR+F+I | - | - | TVM+F+G4 | GTR+F | TVM+F+G4 | - | - |
| *petA* | GTR+F+G4 | TVM+F+I | GTR+F+G4 | - | - | TVM+F+G4 | GTR+F | TVM+F+G4 | - | - |
| *petB* | GTR+F+I | HKY+F+I | GTR+F+G4 | - | - | K3Pu+F+I | GTR+F+I | TVM+F+G4 | - | - |
| *petD* | GTR+F+I | HKY+F+I | GTR+F+G4 | - | - | TVM+F+G4 | GTR+F+I | GTR+F+G4 | - | - |
| *petG* | GTR+F+I | HKY+F+I | HKY+F+I | - | - | TVM+F+I | GTR+F+I | GTR+F+G4 | - | - |
| *petL* | GTR+F+I | TVM+F+I | GTR+F | - | - | K3Pu+F+I | F81+F | TVM+F+G4 | - | - |
| *petN* | HKY+F | HKY+F+I | GTR+F+G4 | - | - | K3Pu+F+I | GTR+F+I | GTR+F+G4 | - | - |
| *psaA* | GTR+F+I | HKY+F+I | GTR+F+G4 | - | - | K3Pu+F+I | GTR+F+I | TVM+F+G4 | - | - |
| *psaB* | GTR+F+I | HKY+F+I | GTR+F | - | - | K3Pu+F+I | GTR+F+I | TVM+F+G4 | - | - |
| *psaC* | GTR+F+I | HKY+F+I | HKY+F+I | - | - | TPM3u+F+I | GTR+F+I | GTR+F+G4 | - | - |
| *psaI* | GTR+F+I | TVM+F+I | GTR+F+G4 | - | - | TVM+F+G4 | F81+F | GTR+F+G4 | - | - |
| *psaJ* | GTR+F+I | K3Pu+F | GTR+F | - | - | K3Pu+F+I | GTR+F | TPM3u+F | - | - |
| *psbA* | GTR+F+I | HKY+F+I | GTR+F | - | - | K3Pu+F+I | GTR+F+I | TVM+F+G4 | - | - |
| *psbB* | GTR+F+I | HKY+F+I | GTR+F | - | - | K3Pu+F+I | GTR+F+I | TVM+F+G4 | - | - |
| *psbC* | GTR+F+I | HKY+F+I | GTR+F+I | - | - | K3Pu+F+I | GTR+F+I | TVM+F+G4 | - | - |
| *psbD* | GTR+F+I | HKY+F+I | GTR+F | - | - | K3Pu+F+I | GTR+F+I | TVM+F+G4 | - | - |
| *psbE* | GTR+F+I | HKY+F+I | GTR+F | - | - | K3Pu+F+I | GTR+F+I | TVM+F+G4 | - | - |
| *psbH* | GTR+F+I | TPM3u+F+I | GTR+F | - | - | TVM+F+G4 | GTR+F+I | TPM3u+F | - | - |
| *psbI* | GTR+F+I | HKY+F+I | GTR+F | - | - | K3Pu+F+I | GTR+F+I | TVM+F+G4 | - | - |
| *psbJ* | GTR+F+I | HKY+F+I | GTR+F | - | - | K3Pu+F+I | GTR+F+I | TPM3u+F | - | - |
| *psbK* | GTR+F+I | TVM+F+I | GTR+F+G4 | - | - | TVM+F+G4 | F81+F | TVM+F+G4 | - | - |
| *psbM* | GTR+F+I | TVM+F+I | GTR+F | - | - | TVM+F+G4 | F81+F | TVM+F+G4 | - | - |
| *psbN* | GTR+F+I | HKY+F+I | GTR+F | - | - | K3Pu+F+I | GTR+F+I | TPM3u+F | - | - |
| *psbT* | GTR+F+I | K3Pu+F | GTR+F+I | - | - | TVM+F+G4 | GTR+F | TVM+F+G4 | - | - |
| *psbZ* | HKY+F | K3Pu+F | GTR+F | - | - | K3Pu+F+I | GTR+F | TVM+F+G4 | - | - |
| *rbcL* | GTR+F+I | TVM+F+I | GTR+F+G4 | - | - | TPM3u+F+I | HKY+F+I | TVM+F+G4 | - | - |
| *rpl14* | GTR+F+G4 | TVM+F+I | GTR+F | - | - | K3Pu+F+I | GTR+F+I | TPM3u+F | - | - |
| *rpl16* | GTR+F | TVM+F+I | GTR+F | - | - | K3Pu+F+I | GTR+F+I | TVM+F+G4 | - | - |
| *rpl2* | F81+F+I | HKY+F+I | GTR+F | - | - | K3Pu+F+I | F81+F | TPM3u+F | - | - |
| *rpl20* | GTR+F+G4 | TVM+F+I | GTR+F | - | - | K3Pu+F+I | GTR+F+I | TVM+F+G4 | - | - |
| *rpl22* | GTR+F+G4 | TPM3u+F+I | GTR+F | - | - | TVM+F+G4 | GTR+F+I | TVM+F+G4 | - | - |
| *rpl23* | F81+F+I | HKY+F+I | GTR+F | - | - | K3Pu+F+I | F81+F | TPM3u+F | - | - |
| *rpl32* | GTR+F+I | TVM+F+I | GTR+F+G4 | - | - | TPM3u+F+I | GTR+F | GTR+F+G4 | - | - |
| *rpl33* | GTR+F+G4 | TVM+F+I | GTR+F+G4 | - | - | TVM+F+G4 | GTR+F | TVM+F+G4 | - | - |
| *rpl36* | GTR+F+G4 | TVM+F+I | GTR+F | - | - | TVM+F+G4 | GTR+F | GTR+F+G4 | - | - |
| *rpoA* | GTR+F+G4 | TVM+F+I | GTR+F | - | - | TVM+F+G4 | GTR+F+I | TVM+F+G4 | - | - |
| *rpoB* | GTR+F+G4 | TVM+F+I | GTR+F | - | - | TVM+F+G4 | GTR+F | TVM+F+G4 | - | - |
| *rpoC1* | GTR+F+G4 | TVM+F+I | GTR+F | - | - | TVM+F+G4 | GTR+F+I | TVM+F+G4 | - | - |
| *rpoC2* | GTR+F+G4 | TVM+F+I | GTR+F | - | - | TVM+F+G4 | GTR+F+I | TVM+F+G4 | - | - |
| *rps11* | K2P | TVM+F+I | GTR+F | - | - | TVM+F+G4 | GTR+F+I | TVM+F+G4 | - | - |
| *rps12* | HKY+F+I | HKY+F+I | GTR+F | - | - | K3Pu+F+I | F81+F | TVM+F+G4 | - | - |
| *rps14* | GTR+F | TVM+F+I | GTR+F | - | - | K3Pu+F+I | HKY+F | TVM+F+G4 | - | - |
| *rps15* | GTR+F+I | TVM+F+I | HKY+F+I | - | - | TPM3u+F+I | HKY+F | GTR+F+G4 | - | - |
| *rps16* | GTR+F | TVM+F+I | GTR+F | - | - | K3Pu+F+I | GTR+F | TVM+F+G4 | - | - |
| *rps18* | GTR+F+G4 | TVM+F+I | GTR+F | - | - | TVM+F+G4 | GTR+F | TVM+F+G4 | - | - |
| *rps19* | GTR+F+I | TVM+F+I | GTR+F+G4 | - | - | TPM3u+F+I | GTR+F | GTR+F+G4 | - | - |
| *rps2* | GTR+F+G4 | TVM+F+I | GTR+F+G4 | - | - | TVM+F+G4 | GTR+F | TVM+F+G4 | - | - |
| *rps3* | GTR+F+G4 | TVM+F+I | GTR+F | - | - | TVM+F+G4 | HKY+F | TVM+F+G4 | - | - |
| *rps4* | GTR+F+G4 | TVM+F+I | GTR+F+I | - | - | TVM+F+G4 | GTR+F+I | TVM+F+G4 | - | - |
| *rps7* | F81+F+I | TVM+F+I | GTR+F | - | - | K3Pu+F+I | GTR+F+I | TPM3u+F | - | - |
| *rps8* | GTR+F+G4 | TPM3u+F+I | GTR+F | - | - | TVM+F+G4 | GTR+F+I | TVM+F+G4 | - | - |
| *ycf1* | GTR+F+G4 | TVM+F+G4 | GTR+F+G4 | - | - | TVM+F+G4 | GTR+F+G4 | GTR+F+G4 | - | - |
| *ycf15* | K2P | TVM+F+I | GTR+F | - | - | TVM+F+G4 | GTR+F+I | TVM+F+G4 | - | - |
| *ycf2* | GTR+F+G4 | TVM+F+I | GTR+F | - | - | K3Pu+F+I | GTR+F | TPM3u+F | - | - |
| *ycf3* | GTR+F+G4 | HKY+F+I | GTR+F | - | - | K3Pu+F+I | F81+F | TVM+F+G4 | - | - |
| *ycf4* | GTR+F+I | TVM+F+I | GTR+F | - | - | K3Pu+F+I | GTR+F+I | TVM+F+G4 | - | - |

**Table S4.** Gene content of plastome of *Ajania*.

| Category | Gene group | Gene name |
| --- | --- | --- |
| Photosynthesis | Subunits of photosystem I | *psaA,psaB,psaC,psaI,psaJ* |
|  | Subunits of photosystem II | *psbA,psbB,psbC,psbD,psbE,psbF,psbH,psbI,psbJ,psbK,psbL,psbM,psbN,psbT,psbZ* |
|  | Subunits of NADH dehydrogenase | *ndhA*,ndhB*(2),ndhC,ndhD,ndhE,ndhF,ndhG,ndhH,ndhI,ndhJ,ndhK* |
|  | Subunits of cytochrome b/f complex | *petA,petB*,petD*,petG,petL,petN* |
|  | Subunits of ATP synthase | *atpA,atpB,atpE,atpF*,atpH,atpI* |
|  | Large subunit of rubisco | *rbcL* |
| Self-replication | Proteins of large ribosomal subunit | *rpl14,rpl16*,rpl2*(2),rpl20,rpl22,rpl23(2),rpl32,rpl33,rpl36* |
|  | Proteins of small ribosomal subunit | *rps11,rps12**(2),rps14,rps15,rps16*,rps18,rps19,rps2,rps3,rps4,rps7(2),rps8* |
|  | Subunits of RNA polymerase | *rpoA,rpoB,rpoC1*,rpoC2* |
|  | Ribosomal RNAs | *rrn16S(2),rrn23S(2),rrn4.5S(2),rrn5S(2)* |
|  | Transfer RNAs | *trnA-UGC*(2),trnC-GCA,trnD-GUC,trnE-UUC,trnF-GAA,trnG-GCC,trnG-UCC*,trnH-GUG,trnI-CAU(2),trnI-GAU*(2),trnK-UUU*,trnL-CAA(2),trnL-UAA*,trnL-UAG,trnM-CAU,trnN-GUU(2),trnP-UGG,trnQ-UUG,trnR-ACG(2),trnR-UCU,trnS-GCU,trnS-GGA(2),trnT-GGU,trnT-UGU,trnV-GAC(2),trnV-UAC*,trnW-CCA,trnY-GUA,trnfM-CAU* |
| Other genes | Maturase | *matK* |
|  | Protease | *clpP*** |
|  | Envelope membrane protein | *cemA* |
|  | Acetyl-CoA carboxylase | *accD* |
|  | c-type cytochrome synthesis gene | *ccsA* |
|  | Translation initiation factor | *infA* |
|  | other | *-* |
| Genes of unknown function | Conserved hypothetical chloroplast ORF | *ycf1,ycf15(2),ycf2(2),ycf3**,ycf4* |

**Table S5.** Sample distribution of long dispersed repeats (LDRs) in the plastome for eight *Ajania* species. F indicates forward repeats, P indicates palindromic repeats, and R indicates reverse repeats.

| Length (bp) | Type | Gene | Region | species |
| --- | --- | --- | --- | --- |
| 60 | F | *ycf2*;*ycf2* | IRb;IRb | *A. fruticulosa* |
| 60 | P | *ycf2*;ycf2*-*2 | IRb;IRa | *A. fruticulosa* |
| 60 | P | *ycf2*;*ycf2*-2 | IRb;IRa | *A. fruticulosa* |
| 60 | F | *ycf2*-2;*ycf2*-2 | IRa;IRa | *A. fruticulosa* |
| 48 | P | *psbN*(partical:4.17%);*psbN*(partical:4.17%) | LSC;LSC | *A. fruticulosa* |
| 45 | F | *ycf2*;*ycf2* | IRb;IRb | *A. fruticulosa* |
| 45 | P | *ycf2*;*ycf2*-2 | IRb;IRa | *A. fruticulosa* |
| 45 | P | *ycf2*;*ycf2*-2 | IRb;IRa | *A. fruticulosa* |
| 39 | F | IGS(*rps12*,*ycf15*);*ndhA*-intron1 | IRb;SSC | *A. fruticulosa* |
| 39 | P | *ndhA*-intron1;IGS(*ycf15*-2,*rps12*-2) | SSC;IRa | *A. fruticulosa* |
| 41 | F | *ycf3*-intron1;IGS(*rps12*,*ycf15*) | LSC;IRb | *A. fruticulosa* |
| 41 | P | *ycf3*-intron1;IGS(*ycf15*-2,*rps12*-2) | LSC;IRa | *A. fruticulosa* |
| 39 | F | *ycf3*-intron1;*ndhA*-intron1 | LSC;SSC | *A. fruticulosa* |
| 42 | F | *ycf2*;*ycf2* | IRb;IRb | *A. fruticulosa* |
| 42 | P | *ycf2*;*ycf2*-2 | IRb;IRa | *A. fruticulosa* |
| 42 | P | *ycf2*;*ycf2*-2 | IRb;IRa | *A. fruticulosa* |
| 42 | F | *ycf2*-2;*ycf2*-2 | IRa;IRa | *A. fruticulosa* |
| 35 | F | IGS(*ycf1*-2,*trnN*-*GUU*-2);IGS(*ycf1*-2,*trnN*-*GUU*-2) | SSC;SSC | *A. fruticulosa* |
| 30 | P | *trnS*-*GGA*(partical:93.33%);*trnS*-*GGA*-2(partical:93.33%) | LSC;LSC | *A. fruticulosa* |
| 30 | F | *ycf2*;*ycf2* | IRb;IRb | *A. fruticulosa* |
| 30 | P | *ycf2*;*ycf2*-2 | IRb;IRa | *A. fruticulosa* |
| 30 | P | *ycf2*;*ycf2*-2 | IRb;IRa | *A. fruticulosa* |
| 30 | F | IGS(*rrn4.5S,rrn5S*);IGS(*rrn4.5S,rrn5S*) | IRb;IRb | *A. fruticulosa* |
| 30 | P | IGS(*rrn4.5S,rrn5S*);IGS(*rrn5S*-2,*rrn4*.*5S*-2) | IRb;IRa | *A. fruticulosa* |
| 30 | P | IGS(*rrn4.5S*,*rrn5S*);IGS(*rrn5S*-2,*rrn4.5S*-2) | IRb;IRa | *A. fruticulosa* |
| 30 | F | IGS(*rrn5S*-2,*rrn4.5S*-2);IGS(*rrn5S*-2,*rrn4.5S*-2) | IRa;IRa | *A. fruticulosa* |
| 35 | F | *ycf3*-intron1;*ndhB*-intron1 | LSC;IRb | *A. fruticulosa* |
| 35 | P | *ycf3*-intron1;*ndhB*-2-intron1 | LSC;IRa | *A. fruticulosa* |
| 32 | F | *psaB*;*psaA* | LSC;LSC | *A. fruticulosa* |
| 33 | F | IGS(*trnK-UUU,rps16*);IGS(*trnK-UUU,rps16*) | LSC;LSC | *A. fruticulosa* |
| 33 | P | IGS(*trnT-GGU,psbD*);IGS(*trnT-GGU,psbD*) | LSC;LSC | *A. fruticulosa* |
| 30 | P | *trnS-GCU*(partical:93.33%);*trnS-GGA*-2(partical:93.33%) | LSC;LSC | *A. fruticulosa* |
| 30 | F | *rbcL*(partical:36.67%);IGS(*rbcL*,*accD*) | LSC;LSC | *A. fruticulosa* |
| 30 | F | IGS(*psaJ*,*rpl33*);IGS(*ycf15*,*trnV-GAC*) | LSC;IRb | *A. fruticulosa* |
| 30 | P | IGS(*psaJ*,*rpl33*);IGS(*trnV-GAC*-2,*ycf15*-2) | LSC;IRa | *A. fruticulosa* |
| 32 | F | *trnS-GGA*;*trnS-GCU* | LSC;LSC | *A. fruticulosa* |
| 30 | F | IGS(*atpF*,*atpA*);IGS(*atpA*,*trnR-UCU*) | LSC;LSC | *A. fruticulosa* |
| 30 | F | *psaB*;*psaA* | LSC;LSC | *A. fruticulosa* |
| 30 | F | *ycf2*;*ycf2*-2 | IRb;IRa | *A. fruticulosa* |
| 60 | F | *ycf2*;*ycf2* | IRb;IRb | *A. ramosa* |
| 60 | P | *ycf2*;*ycf2*-2 | IRb;IRa | *A. ramosa* |
| 60 | P | *ycf2*;*ycf2*-2 | IRb;IRa | *A. ramosa* |
| 60 | F | *ycf2*-2;*ycf2*-2 | IRa;IRa | *A. ramosa* |
| 48 | P | *psbN*;*psbN* | LSC;LSC | *A. ramosa* |
| 45 | F | *ycf2*;*ycf2* | IRb;IRb | *A. ramosa* |
| 45 | P | *ycf2*;*ycf2*-2 | IRb;IRa | *A. ramosa* |
| 45 | P | *ycf2*;*ycf2*-2 | IRb;IRa | *A. ramosa* |
| 39 | F | IGS(*rps12*,*ycf15*);*ndhA*-intron1 | IRb;SSC | *A. ramosa* |
| 39 | P | *ndhA*-intron1;IGS(*ycf15*-2,*rps12*-2) | SSC;IRa | *A. ramosa* |
| 41 | F | *ycf3*-intron1;IGS(*rps12*,*ycf15*) | LSC;IRb | *A. ramosa* |
| 41 | P | *ycf3*-intron1;IGS(*ycf15*-2,*rps12*-2) | LSC;IRa | *A. ramosa* |
| 39 | F | *ycf3*-intron1;*ndhA*-intron1 | LSC;SSC | *A. ramosa* |
| 42 | F | *ycf2*;*ycf2* | IRb;IRb | *A. ramosa* |
| 42 | P | *ycf2*;*ycf2*-2 | IRb;IRa | *A. ramosa* |
| 42 | P | *ycf2*;*ycf2*-2 | IRb;IRa | *A. ramosa* |
| 42 | F | *ycf2*-2;*ycf2*-2 | IRa;IRa | *A. ramosa* |
| 43 | P | *ndhD*;*ndhD* | SSC;SSC | *A. ramosa* |
| 30 | P | *trnS*-*GGA*;*trnS*-*GGA-*2 | LSC;LSC | *A. ramosa* |
| 30 | F | *ycf2*;*ycf2* | IRb;IRb | *A. ramosa* |
| 30 | P | *ycf2*;*ycf2*-2 | IRb;IRa | *A. ramosa* |
| 30 | P | *ycf2*;*ycf2*-2 | IRb;IRa | *A. ramosa* |
| 30 | F | IGS(*rrn4*.*5S*,*rrn5S*);IGS(*rrn4.5S*,*rrn5S*) | IRb;IRb | *A. ramosa* |
| 30 | P | IGS(*rrn4.5S*,*rrn5S*);IGS(*rrn5S*-2,*rrn4.5S*-2) | IRb;IRa | *A. ramosa* |
| 30 | P | IGS(*rrn4.5S*,*rrn5S*);IGS(*rrn5S*-2,*rrn4.5S*-2) | IRb;IRa | *A. ramosa* |
| 30 | F | IGS(*rrn5S*-2,*rrn4.5S*-2);IGS(*rrn5S*-2,*rrn4.5S*-2) | IRa;IRa | *A. ramosa* |
| 35 | F | *ycf3*-intron1;*ndhB*-intron1 | LSC;IRb | *A. ramosa* |
| 35 | P | *ycf3*-intron1;*ndhB*-2-intron1 | LSC;IRa | *A. ramosa* |
| 32 | F | *psaB*;*psaA* | LSC;LSC | *A. ramosa* |
| 31 | R | IGS(*psbI*,*trnS*-*GGA*);IGS(*psbI*,*trnS*-*GGA*) | LSC;LSC | *A. ramosa* |
| 33 | F | IGS(*trnK*-*UUU*,*rps16*);IGS(*trnK*-*UUU*,*rps16*) | LSC;LSC | *A. ramosa* |
| 30 | P | *trnS*-*GCU*;*trnS*-*GGA*-2 | LSC;LSC | *A. ramosa* |
| 30 | F | IGS(*psaJ*,*rpl33*);IGS(*ycf15*,*trnV*-*GAC*) | LSC;IRb | *A. ramosa* |
| 30 | P | IGS(*psaJ*,*rpl33*);IGS(*trnV*-*GAC*-2,*ycf15*-2) | LSC;IRa | *A. ramosa* |
| 32 | F | *trnS*-*GGA*;*trnS*-*GCU* | LSC;LSC | *A. ramosa* |
| 30 | R | IGS(*atpA*,*trnR*-*UCU*);*clpP*-intron2 | LSC;LSC | *A. ramosa* |
| 30 | F | *psaB*;*psaA* | LSC;LSC | *A. ramosa* |
| 30 | F | *ycf2*;*ycf2*-2 | IRb;IRa | *A. ramosa* |
| 60 | F | *ycf2*;*ycf2* | IRb;IRb | *A. przewalskii* |
| 60 | P | *ycf2*;*ycf2*-2 | IRb;IRa | *A. przewalskii* |
| 60 | P | *ycf2*;*ycf2*-2 | IRb;IRa | *A. przewalskii* |
| 60 | F | *ycf2*-2;*ycf2*-2 | IRa;IRa | *A. przewalskii* |
| 48 | P | *psbN*;*psbN* | LSC;LSC | *A. przewalskii* |
| 45 | F | *ycf2*;*ycf2* | IRb;IRb | *A. przewalskii* |
| 45 | P | *ycf2*;*ycf2*-2 | IRb;IRa | *A. przewalskii* |
| 45 | P | *ycf2*;*ycf2*-2 | IRb;IRa | *A. przewalskii* |
| 43 | F | IGS(*trnY*-*GUA*,*trnE*-*UUC*);IGS(*trnY*-*GUA*,*trnE*-*UUC*) | LSC;LSC | *A. przewalskii* |
| 39 | F | IGS(*rps12*,*ycf15*);*ndhA*-intron1 | IRb;SSC | *A. przewalskii* |
| 39 | P | *ndhA*-intron1;IGS(*ycf15*-2,*rps12*-2) | SSC;IRa | *A. przewalskii* |
| 41 | F | *ycf3*-intron1;IGS(*rps12*,*ycf15*) | LSC;IRb | *A. przewalskii* |
| 41 | P | *ycf3*-intron1;IGS(*ycf15*-2,*rps12*-2) | LSC;IRa | *A. przewalskii* |
| 39 | F | *ycf3*-intron1;*ndhA*-intron1 | LSC;SSC | *A. przewalskii* |
| 42 | F | *ycf2*;*ycf2* | IRb;IRb | *A. przewalskii* |
| 42 | P | *ycf2*;*ycf2*-2 | IRb;IRa | *A. przewalskii* |
| 42 | P | *ycf2*;*ycf2*-2 | IRb;IRa | *A. przewalskii* |
| 42 | F | *ycf2*-2;*ycf2*-2 | IRa;IRa | *A. przewalskii* |
| 35 | F | IGS(*ycf1*-2,*trnN*-*GUU*-2);IGS(*ycf1*-2,*trnN*-*GUU*-2) | SSC;SSC | *A. przewalskii* |
| 30 | P | *trnS*-*GGA*;*trnS*-*GGA*-2 | LSC;LSC | *A. przewalskii* |
| 30 | F | *ycf2*;*ycf2* | IRb;IRb | *A. przewalskii* |
| 30 | P | *ycf2*;*ycf2*-2 | IRb;IRa | *A. przewalskii* |
| 30 | P | *ycf2*;*ycf2*-2 | IRb;IRa | *A. przewalskii* |
| 30 | F | IGS(*rrn4.5S*,*rrn5S*);IGS(*rrn4.5S*,*rrn5S*) | IRb;IRb | *A. przewalskii* |
| 30 | P | IGS(*rrn4.5S*,*rrn5S*);IGS(*rrn5S*-2*,rrn4.5S*-2) | IRb;IRa | *A. przewalskii* |
| 30 | P | IGS(*rrn4.5S*,*rrn5S*);IGS(*rrn5S*-2,*rrn4.5S*-2) | IRb;IRa | *A. przewalskii* |
| 30 | F | IGS(*rrn5S*-2,*rrn4*.*5S*-2);IGS(*rrn5S*-2,*rrn4.5S*-2) | IRa;IRa | *A. przewalskii* |
| 35 | F | *ycf3*-intron1;*ndhB*-intron1 | LSC;IRb | *A. przewalskii* |
| 35 | P | *ycf3*-intron1;*ndhB*-2-intron1 | LSC;IRa | *A. przewalskii* |
| 32 | F | *psaB*;*psaA* | LSC;LSC | *A. przewalskii* |
| 33 | F | IGS(*trnK*-*UUU*,*rps16*);IGS(*trnK*-*UUU*,*rps16*) | LSC;LSC | *A. przewalskii* |
| 30 | P | *trnS*-*GCU*;*trnS*-*GGA*-2 | LSC;LSC | *A. przewalskii* |
| 30 | F | *rbcL*;IGS(*rbcL*,*accD*) | LSC;LSC | *A. przewalskii* |
| 30 | F | IGS(*psaJ*,*rpl33*);IGS(*ycf15*,*trnV-GAC*) | LSC;IRb | *A. przewalskii* |
| 30 | P | IGS(*psaJ*,*rpl33*);IGS(*trnV*-*GAC*-2,*ycf15*-2) | LSC;IRa | *A. przewalskii* |
| 32 | F | *trnS*-*GGA*;*trnS*-*GCU* | LSC;LSC | *A. przewalskii* |
| 30 | F | *psaB*;*psaA* | LSC;LSC | *A. przewalskii* |
| 30 | F | *ycf2*;*ycf2*-2 | IRb;IRa | *A. przewalskii* |
| 60 | F | *ycf2*;*ycf2* | IRb;IRb | *A. nematoloba* |
| 60 | P | *ycf2*;*ycf2*-2 | IRb;IRa | *A. nematoloba* |
| 60 | P | *ycf2*;*ycf2*-2 | IRb;IRa | *A. nematoloba* |
| 60 | F | *ycf2*-2;*ycf2*-2 | IRa;IRa | *A. nematoloba* |
| 48 | P | *psbN*;*psbN* | LSC;LSC | *A. nematoloba* |
| 45 | F | *ycf2*;*ycf2* | IRb;IRb | *A. nematoloba* |
| 45 | P | *ycf2*;*ycf2*-2 | IRb;IRa | *A. nematoloba* |
| 45 | P | *ycf2*;*ycf2*-2 | IRb;IRa | *A. nematoloba* |
| 39 | F | IGS(*rps12*,*ycf15*);*ndhA*-intron1 | IRb;SSC | *A. nematoloba* |
| 39 | P | *ndhA*-intron1;IGS(*ycf15*-2,*rps12*-2) | SSC;IRa | *A. nematoloba* |
| 41 | F | *ycf3*-intron1;IGS(*rps12*,*ycf15*) | LSC;IRb | *A. nematoloba* |
| 41 | P | *ycf3*-intron1;IGS(*ycf15*-2,*rps12*-2) | LSC;IRa | *A. nematoloba* |
| 39 | F | *ycf3*-intron1;*ndhA*-intron1 | LSC;SSC | *A. nematoloba* |
| 42 | F | *ycf2*;*ycf2* | IRb;IRb | *A. nematoloba* |
| 42 | P | *ycf2*;*ycf2*-2 | IRb;IRa | *A. nematoloba* |
| 42 | P | *ycf2*;*ycf2*-2 | IRb;IRa | *A. nematoloba* |
| 42 | F | *ycf2*-2;*ycf2*-2 | IRa;IRa | *A. nematoloba* |
| 35 | F | IGS(*ycf1*-2,*trnN*-*GUU*-2);IGS(*ycf1*-2,trnN-*GUU*-2) | SSC;SSC | *A. nematoloba* |
| 34 | F | *rps16*-intron1;*rps16*-intron1 | LSC;LSC | *A. nematoloba* |
| 30 | R | *rps16*-intron1;*rps16*-intron1 | LSC;LSC | *A. nematoloba* |
| 30 | P | *trnS*-*GGA*;*trnS*-*GGA*-2 | LSC;LSC | *A. nematoloba* |
| 30 | F | *ycf2*;*ycf2* | IRb;IRb | *A. nematoloba* |
| 30 | P | *ycf2*;*ycf2*-2 | IRb;IRa | *A. nematoloba* |
| 30 | P | *ycf2*;*ycf2*-2 | IRb;IRa | *A. nematoloba* |
| 30 | F | IGS(*rrn4.5S*,*rrn5S*);IGS(*rrn4.5S*,*rrn5S*) | IRb;IRb | *A. nematoloba* |
| 30 | P | IGS(*rrn4.5S*,*rrn5S*);IGS(*rrn5S*-2,*rrn4.5S*-2) | IRb;IRa | *A. nematoloba* |
| 30 | P | IGS(*rrn4.5S*,*rrn5S*);IGS(*rrn5S*-2,*rrn4.5S*-2) | IRb;IRa | *A. nematoloba* |
| 30 | F | IGS(*rrn5S*-2,*rrn4.5S*-2);IGS(*rrn5S*-2,*rrn4.5S*-2) | IRa;IRa | *A. nematoloba* |
| 35 | P | IGS(*psaA*,*ycf3*);IGS(*psaA*,*ycf3*) | LSC;LSC | *A. nematoloba* |
| 35 | F | *ycf3*-intron1;*ndhB*-intron1 | LSC;IRb | *A. nematoloba* |
| 35 | P | *ycf3*-intron1;*ndhB*-2-intron1 | LSC;IRa | *A. nematoloba* |
| 32 | R | *rps16*-intron1;*rps16*-intron1 | LSC;LSC | *A. nematoloba* |
| 32 | F | *psaB*;*psaA* | LSC;LSC | *A. nematoloba* |
| 30 | P | *trnS*-*GCU*;*trnS*-*GGA*-2 | LSC;LSC | *A. nematoloba* |
| 30 | F | *rbcL*;IGS(*rbcL*,*accD*) | LSC;LSC | *A. nematoloba* |
| 30 | F | IGS(*psaJ*,*rpl33*);IGS(*ycf15*,*trnV*-*GAC*) | LSC;IRb | *A. nematoloba* |
| 30 | P | IGS(*psaJ*,*rpl33*);IGS(*trnV*-*GAC*-2,*ycf15*-2) | LSC;IRa | *A. nematoloba* |
| 32 | F | *trnS*-*GGA*;*trnS*-*GCU* | LSC;LSC | *A. nematoloba* |
| 31 | F | IGS(*psbE*,*petL*);IGS(*psbE*,*petL*) | LSC;LSC | *A. nematoloba* |
| 30 | F | *psaB*;*psaA* | LSC;LSC | *A. nematoloba* |
| 30 | F | IGS(*accD*,*psaI*);IGS(*accD*,*psaI*) | LSC;LSC | *A. nematoloba* |
| 30 | F | *ycf2*;*ycf2*-2 | IRb;IRa | *A. nematoloba* |
| 60 | F | *ycf2*;*ycf2* | IRb;IRb | *A. khartensis* |
| 60 | P | *ycf2*;*ycf2*-2 | IRb;IRa | *A. khartensis* |
| 60 | P | *ycf2*;*ycf2*-2 | IRb;IRa | *A. khartensis* |
| 60 | F | *ycf2*-2;*ycf2*-2 | IRa;IRa | *A. khartensis* |
| 48 | P | *psbN*;*psbN* | LSC;LSC | *A. khartensis* |
| 45 | F | *ycf2*;*ycf2* | IRb;IRb | *A. khartensis* |
| 45 | P | *ycf2*;*ycf2*-2 | IRb;IRa | *A. khartensis* |
| 45 | P | *ycf2*;*ycf2*-2 | IRb;IRa | *A. khartensis* |
| 43 | F | IGS(*trnY*-*GUA*,*trnE*-*UUC*);IGS(*trnY*-*GUA*,*trnE*-*UUC*) | LSC;LSC | *A. khartensis* |
| 39 | F | IGS(*rps12*,*ycf15*);*ndhA*-intron1 | IRb;SSC | *A. khartensis* |
| 39 | P | *ndhA*-intron1;IGS(*ycf15*-2,*rps12*-2) | SSC;IRa | *A. khartensis* |
| 41 | F | *ycf3*-intron1;IGS(*rps12*,*ycf15*) | LSC;IRb | *A. khartensis* |
| 41 | P | *ycf3*-intron1;IGS(*ycf15*-2,*rps12*-2) | LSC;IRa | *A. khartensis* |
| 39 | F | *ycf3*-intron1;*ndhA*-intron1 | LSC;SSC | *A. khartensis* |
| 42 | F | *ycf2*;*ycf2* | IRb;IRb | *A. khartensis* |
| 42 | P | *ycf2*;*ycf2*-2 | IRb;IRa | *A. khartensis* |
| 42 | P | *ycf2*;*ycf2*-2 | IRb;IRa | *A. khartensis* |
| 42 | F | *ycf2*-2;*ycf2*-2 | IRa;IRa | *A. khartensis* |
| 35 | F | IGS(*ycf1*-2,*trnN*-*GUU*-2);IGS(*ycf1*-2,*trnN*-*GUU*-2) | SSC;SSC | *A. khartensis* |
| 30 | P | *trnS*-*GGA*;*trnS*-*GGA*-2 | LSC;LSC | *A. khartensis* |
| 30 | F | *ycf2*;*ycf2* | IRb;IRb | *A. khartensis* |
| 30 | P | *ycf2*;*ycf2*-2 | IRb;IRa | *A. khartensis* |
| 30 | P | *ycf2*;*ycf2*-2 | IRb;IRa | *A. khartensis* |
| 30 | F | IGS(*rrn4*.*5S*,*rrn5S*);IGS(*rrn4*.*5S*,*rrn5S*) | IRb;IRb | *A. khartensis* |
| 30 | P | IGS(*rrn4*.*5S*,*rrn5S*);IGS(*rrn5S*-2,*rrn4*.*5S*-2) | IRb;IRa | *A. khartensis* |
| 30 | P | IGS(*rrn4.5S*,*rrn5S*);IGS(*rrn5S*-2,*rrn4.5S*-2) | IRb;IRa | *A. khartensis* |
| 30 | F | IGS(*rrn5S*-2,*rrn4.5S*-2);IGS(*rrn5S*-2,*rrn4.5S*-2) | IRa;IRa | *A. khartensis* |
| 35 | F | *ycf3*-intron1;*ndhB*-intron1 | LSC;IRb | *A. khartensis* |
| 35 | P | *ycf3*-intron1;*ndhB*-2-intron1 | LSC;IRa | *A. khartensis* |
| 32 | F | *psaB*;*psaA* | LSC;LSC | *A. khartensis* |
| 33 | F | IGS(*trnK*-*UUU*,*rps16*);IGS(*trnK*-*UUU*,*rps16*) | LSC;LSC | *A. khartensis* |
| 30 | P | *trnS*-*GCU*;*trnS*-*GGA*-2 | LSC;LSC | *A. khartensis* |
| 30 | F | *rbcL*;IGS(*rbcL*,*accD*) | LSC;LSC | *A. khartensis* |
| 30 | F | IGS(*psaJ*,*rpl33*);IGS(*ycf15*,*trnV*-*GAC*) | LSC;IRb | *A. khartensis* |
| 30 | P | IGS(*psaJ*,*rpl33*);IGS(*trnV*-*GAC*-2,*ycf15*-2) | LSC;IRa | *A. khartensis* |
| 32 | F | *trnS*-*GGA*;*trnS*-*GCU* | LSC;LSC | *A. khartensis* |
| 30 | F | *psaB*;*psaA* | LSC;LSC | *A. khartensis* |
| 30 | F | *ycf2*;*ycf2*-2 | IRb;IRa | *A. khartensis* |
| 60 | F | *ycf2*;*ycf2* | IRb;IRb | *A. variifolia* |
| 60 | P | *ycf2*;*ycf2*-2 | IRb;IRa | *A. variifolia* |
| 60 | P | *ycf2*;*ycf2*-2 | IRb;IRa | *A. variifolia* |
| 60 | F | *ycf2*-2;*ycf2*-2 | IRa;IRa | *A. variifolia* |
| 48 | P | *psbN*;*psbN* | LSC;LSC | *A. variifolia* |
| 45 | F | *ycf2*;*ycf2* | IRb;IRb | *A. variifolia* |
| 45 | P | *ycf2*;*ycf2*-2 | IRb;IRa | *A. variifolia* |
| 45 | P | *ycf2*;*ycf2*-2 | IRb;IRa | *A. variifolia* |
| 39 | F | IGS(*rps12*,*ycf15*);*ndhA*-intron1 | IRb;SSC | *A. variifolia* |
| 39 | P | *ndhA*-intron1;IGS(*ycf15*-2,*rps12*-2) | SSC;IRa | *A. variifolia* |
| 41 | F | *ycf3*-intron1;IGS(*rps12*,ycf15) | LSC;IRb | *A. variifolia* |
| 41 | P | *ycf3*-intron1;IGS(*ycf15*-2,*rps12*-2) | LSC;IRa | *A. variifolia* |
| 39 | F | *ycf3*-intron1;*ndhA*-intron1 | LSC;SSC | *A. variifolia* |
| 42 | F | *ycf2*;*ycf2* | IRb;IRb | *A. variifolia* |
| 42 | P | *ycf2*;*ycf2*-2 | IRb;IRa | *A. variifolia* |
| 42 | P | *ycf2*;*ycf2*-2 | IRb;IRa | *A. variifolia* |
| 42 | F | *ycf2*-2;*ycf2*-2 | IRa;IRa | *A. variifolia* |
| 43 | P | *ndhD*;*ndhD* | SSC;SSC | *A. variifolia* |
| 33 | F | IGS(*trnK*-*UUU*,*rps16*);IGS(*trnK*-*UUU*,*rps16*) | LSC;LSC | *A. variifolia* |
| 31 | F | IGS(*trnT*-*GGU*,*psbD*);IGS(*trnT*-*GGU*,*psbD*) | LSC;LSC | *A. variifolia* |
| 30 | P | *trnS*-*GGA*;*trnS*-*GGA*-2 | LSC;LSC | *A. variifolia* |
| 30 | F | *ycf2*;*ycf2* | IRb;IRb | *A. variifolia* |
| 30 | P | *ycf2*;*ycf2*-2 | IRb;IRa | *A. variifolia* |
| 30 | P | *ycf2*;*ycf2*-2 | IRb;IRa | *A. variifolia* |
| 30 | F | IGS(*rrn4.5S*,*rrn5S*);IGS(*rrn4.5S*,*rrn5S*) | IRb;IRb | *A. variifolia* |
| 30 | P | IGS(*rrn4.5S*,*rrn5S*);IGS(*rrn5S*-2,*rrn4.5S*-2) | IRb;IRa | *A. variifolia* |
| 30 | P | IGS(*rrn4.5S*,*rrn5S*);IGS(*rrn5S*-2,*rrn4.5S*-2) | IRb;IRa | *A. variifolia* |
| 30 | F | IGS(*rrn5S*-2,*rrn4.5S*-2);IGS(*rrn5S*-2,*rrn4.5S*-2) | IRa;IRa | *A. variifolia* |
| 35 | F | *ycf3*-intron1;*ndhB*-intron1 | LSC;IRb | *A. variifolia* |
| 35 | P | *ycf3*-intron1;*ndhB*-2-intron1 | LSC;IRa | *A. variifolia* |
| 32 | F | *psaB*;*psaA* | LSC;LSC | *A. variifolia* |
| 30 | P | *trnS*-*GCU*;*trnS-GGA*-2 | LSC;LSC | *A. variifolia* |
| 32 | F | *trnS*-*GGA*;*trnS*-*GCU* | LSC;LSC | *A. variifolia* |
| 31 | F | IGS(*trnT*-*GGU*,*psbD*);IGS(*trnT*-*GGU*,*psbD*) | LSC;LSC | *A. variifolia* |
| 31 | P | IGS(*trnT*-*GGU*,*psbD*);IGS(*psaA*,*ycf3*) | LSC;LSC | *A. variifolia* |
| 30 | F | *psaB*;*psaA* | LSC;LSC | *A. variifolia* |
| 30 | F | *ycf2*;*ycf2*-2 | IRb;IRa | *A. variifolia* |
| 60 | F | *ycf2*;*ycf2* | IRb;IRb | *A. pacifica* |
| 60 | P | *ycf2*;*ycf2*-2 | IRb;IRa | *A. pacifica* |
| 60 | P | *ycf2*;*ycf2*-2 | IRb;IRa | *A. pacifica* |
| 60 | F | *ycf2*-2;*ycf2*-2 | IRa;IRa | *A. pacifica* |
| 48 | P | *psbN*;*psbN* | LSC;LSC | *A. pacifica* |
| 45 | F | *ycf2*;*ycf2* | IRb;IRb | *A. pacifica* |
| 45 | P | *ycf2*;*ycf2*-2 | IRb;IRa | *A. pacifica* |
| 45 | P | *ycf2*;*ycf2*-2 | IRb;IRa | *A. pacifica* |
| 39 | F | IGS(*rps12*,*ycf15*);*ndhA*-intron1 | IRb;SSC | *A. pacifica* |
| 39 | P | *ndhA*-intron1;IGS(*ycf15*-2,*rps12*-2) | SSC;IRa | *A. pacifica* |
| 41 | F | *ycf3*-intron1;IGS(*rps12*,*ycf15*) | LSC;IRb | *A. pacifica* |
| 41 | P | *ycf3*-intron1;IGS(*ycf15*-2,*rps12-*2) | LSC;IRa | *A. pacifica* |
| 39 | F | ycf3-intron1;*ndhA*-intron1 | LSC;SSC | *A. pacifica* |
| 42 | F | *ycf2*;*ycf2* | IRb;IRb | *A. pacifica* |
| 42 | P | *ycf2*;*ycf2*-2 | IRb;IRa | *A. pacifica* |
| 42 | P | *ycf2*;*ycf2*-2 | IRb;IRa | *A. pacifica* |
| 42 | F | *ycf2*-2;*ycf2*-2 | IRa;IRa | *A. pacifica* |
| 41 | P | *ndhD*;*ndhD* | SSC;SSC | *A. pacifica* |
| 30 | P | *trnS*-*GGA*;*trnS*-*GGA*-2 | LSC;LSC | *A. pacifica* |
| 30 | F | *ycf2*;*ycf2* | IRb;IRb | *A. pacifica* |
| 30 | P | *ycf2*;*ycf2*-2 | IRb;IRa | *A. pacifica* |
| 30 | P | *ycf2*;*ycf2*-2 | IRb;IRa | *A. pacifica* |
| 30 | F | IGS(*rrn4.5S*,*rrn5S*);IGS(*rrn4.5S*,*rrn5S*) | IRb;IRb | *A. pacifica* |
| 30 | P | IGS(*rrn4.5S*,*rrn5S*);IGS(*rrn5S*-2,*rrn4.5S*-2) | IRb;IRa | *A. pacifica* |
| 30 | P | IGS(*rrn4.5S*,*rrn5S*);IGS(*rrn5S*-2,*rrn4.5S*-2) | IRb;IRa | *A. pacifica* |
| 30 | F | IGS(*rrn5S*-2,*rrn4.5S*-2);IGS(*rrn5S*-2,*rrn4.5S*-2) | IRa;IRa | *A. pacifica* |
| 35 | F | *ycf3*-intron1;*ndhB*-intron1 | LSC;IRb | *A. pacifica* |
| 35 | P | *ycf3*-intron1;*ndhB*-2-intron1 | LSC;IRa | *A. pacifica* |
| 32 | F | *psaB*;*psaA* | LSC;LSC | *A. pacifica* |
| 33 | F | IGS(*trnK*-*UUU*,*rps16*);IGS(*trnK*-*UUU*,*rps16*) | LSC;LSC | *A. pacifica* |
| 30 | F | IGS(*atpF*,*atpA*);IGS(*atpA*,*trnR*-*UCU*) | LSC;LSC | *A. pacifica* |
| 30 | P | *trnS*-*GCU*;*trnS*-*GGA*-2 | LSC;LSC | *A. pacifica* |
| 30 | F | *rbcL*;IGS(*rbcL*,*accD*) | LSC;LSC | *A. pacifica* |
| 30 | F | IGS(*psaJ*,*rpl33*);IGS(*ycf15*,*trnV*-*GAC*) | LSC;IRb | *A. pacifica* |
| 30 | P | IGS(*psaJ*,*rpl33*);IGS(*trnV*-*GAC*-2,*ycf15*-2) | LSC;IRa | *A. pacifica* |
| 32 | F | *trnS*-*GGA*;*trnS*-*GCU* | LSC;LSC | *A. pacifica* |
| 30 | F | *psaB*;*psaA* | LSC;LSC | *A. pacifica* |
| 30 | F | *ycf2*;*ycf2*-2 | IRb;IRa | *A. pacifica* |
| 60 | F | *ycf2*;*ycf2* | IRb;IRb | *A. tenuifolia* |
| 60 | P | *ycf2*;*ycf2*-2 | IRb;IRa | *A. tenuifolia* |
| 60 | P | *ycf2*;*ycf2*-2 | IRb;IRa | *A. tenuifolia* |
| 60 | F | *ycf2*-2;*ycf2*-2 | IRa;IRa | *A. tenuifolia* |
| 48 | P | *psbN*;*psbN* | LSC;LSC | *A. tenuifolia* |
| 45 | F | *ycf2*;*ycf2* | IRb;IRb | *A. tenuifolia* |
| 45 | P | *ycf2*;*ycf2*-2 | IRb;IRa | *A. tenuifolia* |
| 45 | P | *ycf2*;*ycf2*-2 | IRb;IRa | *A. tenuifolia* |
| 39 | F | IGS(*rps12*,*ycf15*);*ndhA*-intron1 | IRb;SSC | *A. tenuifolia* |
| 39 | P | *ndhA*-intron1;IGS(*ycf15*-2,*rps12*-2) | SSC;IRa | *A. tenuifolia* |
| 41 | F | *ycf3*-intron1;IGS(*rps12*,*ycf15*) | LSC;IRb | *A. tenuifolia* |
| 41 | P | *ycf3*-intron1;IGS(*ycf15*-2,*rps12*-2) | LSC;IRa | *A. tenuifolia* |
| 39 | F | *ycf3*-intron1;*ndhA*-intron1 | LSC;SSC | *A. tenuifolia* |
| 42 | F | *ycf2*;*ycf2* | IRb;IRb | *A. tenuifolia* |
| 42 | P | *ycf2*;*ycf2*-2 | IRb;IRa | *A. tenuifolia* |
| 42 | P | *ycf2*;*ycf2*-2 | IRb;IRa | *A. tenuifolia* |
| 42 | F | *ycf2*-2;*ycf2*-2 | IRa;IRa | *A. tenuifolia* |
| 35 | F | IGS(*ycf1*-2,*trnN*-*GUU*-2);IGS(*ycf1*-2,*trnN*-*GUU*-2) | SSC;SSC | *A. tenuifolia* |
| 30 | P | *trnS*-*GGA*;*trnS*-*GGA*-2 | LSC;LSC | *A. tenuifolia* |
| 30 | F | *ycf2*;*ycf2* | IRb;IRb | *A. tenuifolia* |
| 30 | P | *ycf2*;*ycf2*-2 | IRb;IRa | *A. tenuifolia* |
| 30 | P | *ycf2*;*ycf2*-2 | IRb;IRa | *A. tenuifolia* |
| 30 | F | IGS(*rrn4.5S*,*rrn5S*);IGS(*rrn4.5S*,*rrn5S*) | IRb;IRb | *A. tenuifolia* |
| 30 | P | IGS(*rrn4.5S*,*rrn5S*);IGS(*rrn5S*-2,*rrn4.5S*-2) | IRb;IRa | *A. tenuifolia* |
| 30 | P | IGS(*rrn4.5S*,*rrn5S*);IGS(*rrn5S*-2,*rrn4.5S*-2) | IRb;IRa | *A. tenuifolia* |
| 30 | F | IGS(*rrn5S*-2,*rrn4.5S*-2);IGS(*rrn5S*-2,*rrn4.5S*-2) | IRa;IRa | *A. tenuifolia* |
| 35 | F | *ycf3*-intron1;*ndhB*-intron1 | LSC;IRb | *A. tenuifolia* |
| 35 | P | *ycf3*-intron1;*ndhB*-2-intron1 | LSC;IRa | *A. tenuifolia* |
| 32 | F | *psaB*;*psaA* | LSC;LSC | *A. tenuifolia* |
| 33 | F | IGS(*trnK*-*UUU*,*rps16*);IGS(*trnK*-*UUU*,*rps16*) | LSC;LSC | *A. tenuifolia* |
| 30 | P | *trnS*-*GCU*;*trnS*-*GGA*-2 | LSC;LSC | *A. tenuifolia* |
| 30 | F | *rbcL*(partical:36.67%);IGS(*rbcL*,*accD*) | LSC;LSC | *A. tenuifolia* |
| 30 | F | IGS(*psaJ*,*rpl33*);IGS(*ycf15*,*trnV*-*GAC*) | LSC;IRb | *A. tenuifolia* |
| 30 | P | IGS(*psaJ*,*rpl33*);IGS(*trnV*-*GAC*-2,*ycf15*-2) | LSC;IRa | *A. tenuifolia* |
| 32 | F | *trnS*-*GGA*;*trnS*-*GCU* | LSC;LSC | *A. tenuifolia* |
| 31 | F | IGS(*psbE*,*petL*);IGS(*psbE*,*petL*) | LSC;LSC | *A. tenuifolia* |
| 30 | R | IGS(*atpA*,*trnR*-*UCU*);*clpP*-intron2 | LSC;LSC | *A. tenuifolia* |
| 30 | F | *psaB*;*psaA* | LSC;LSC | *A. tenuifolia* |
| 30 | F | *ycf2*;*ycf2*-2 | IRb;IRa | *A. tenuifolia* |

**Table S6.** Sample distribution of simple repeat sequence (SSR) in the plastome for eight *Ajania* species. p1 indicates single nucleotide repeats, p2 indicates dinucleotide repeats, p3 indicates trinucleotide repeats, p4 indicates tetranucleotide repeats, p5 indicates pentanucleotide repeats, p6 indicates hexanucleotide repeats, and c indicates complex repeats.

| SSR type | size (bp) | ID | location | Region |
| --- | --- | --- | --- | --- |
| p2 | 10 | *A. fruticulosa* | *rpoC1*-*exon2* | LSC |
| p2 | 10 | *A. fruticulosa* | IGS(*trnG*-*UCC*,*trnT*-*GGU*) | LSC |
| p2 | 10 | *A. fruticulosa* | IGS(*psbZ*,*trnG*-*GCC*) | LSC |
| p2 | 10 | *A. fruticulosa* | IGS(*psaA*,*ycf3*) | LSC |
| p2 | 10 | *A. fruticulosa* | *ycf1* | SSC |
| p1 | 10 | *A. fruticulosa* | IGS(*trnK*-*UUU*,*rps16*) | LSC |
| p1 | 10 | *A. fruticulosa* | IGS(*psbM*,*trnD*-*GUC*) | LSC |
| p1 | 10 | *A. fruticulosa* | *rpoB* | LSC |
| p1 | 10 | *A. fruticulosa* | *rpoC1*-exon2 | LSC |
| p1 | 10 | *A. fruticulosa* | *rpoC1*-exon2 | LSC |
| p1 | 10 | *A. fruticulosa* | IGS(*rpoC2*,*rps2*) | LSC |
| p1 | 10 | *A. fruticulosa* | IGS(*atpI*,*atpH*) | LSC |
| p1 | 10 | *A. fruticulosa* | IGS(*psbZ*,*trnG*-GCC) | LSC |
| p1 | 10 | *A. fruticulosa* | IGS(*trnT*-UGU,trnL-*UAA*) | LSC |
| p1 | 10 | *A. fruticulosa* | *rpoA* | LSC |
| p1 | 10 | *A. fruticulosa* | IGS(*rps19*,*rpl2*) | IRb |
| p1 | 10 | *A. fruticulosa* | IGS(*rrn5S*,*trnR*-*ACG*) | IRb |
| p1 | 10 | *A. fruticulosa* | IGS(*trnR*-ACG-2,rrn5S-2) | IRa |
| p1 | 10 | *A. fruticulosa* | IGS(*rpl2*-2,*trnH*-*GUG*) | IRa |
| p1 | 11 | *A. fruticulosa* | IGS(*trnR*-*UCU*,*trnG*-*UCC*) | LSC |
| p1 | 11 | *A. fruticulosa* | IGS(*psbC*,*trnS*-*GCU*) | LSC |
| p1 | 11 | *A. fruticulosa* | *ycf3*-intron1 | LSC |
| p1 | 11 | *A. fruticulosa* | IGS(*atpB*,*rbcL*) | LSC |
| p1 | 11 | *A. fruticulosa* | IGS(*rbcL*,*accD*) | LSC |
| p1 | 11 | *A. fruticulosa* | IGS(*petA*,*psbJ*) | LSC |
| p1 | 11 | *A. fruticulosa* | IGS(*rps18*,*rpl20*) | LSC |
| p1 | 11 | *A. fruticulosa* | *trnI*-*GAU*-intron1 | IRb |
| p1 | 11 | *A. fruticulosa* | IGS(*ndhF*,*rpl32*) | SSC |
| p1 | 11 | *A. fruticulosa* | *ycf1*-2 | SSC |
| p1 | 11 | *A. fruticulosa* | *trnI*-*GAU*-2-intron1 | IRa |
| p4 | 12 | *A. fruticulosa* | IGS(*rps16*,*trnQ*-*UUG*) | LSC |
| p4 | 12 | *A. fruticulosa* | psbM | LSC |
| p4 | 12 | *A. fruticulosa* | IGS(*trnS*-*GCU*,*psbZ*) | LSC |
| p4 | 12 | *A. fruticulosa* | *trnL*-*UAA*-intron1 | LSC |
| p4 | 12 | *A. fruticulosa* | *ycf2* | IRb |
| p4 | 12 | *A. fruticulosa* | IGS(*rpl32*,*trnL*-*UAG*) | SSC |
| p4 | 12 | *A. fruticulosa* | *ndhD* | SSC |
| p4 | 12 | *A. fruticulosa* | *ndhA*-intron1 | SSC |
| p4 | 12 | *A. fruticulosa* | *ycf2-2* | IRa |
| p3 | 12 | *A. fruticulosa* | *psbC* | LSC |
| p1 | 12 | *A. fruticulosa* | IGS(*ycf4*,*cemA*) | LSC |
| p1 | 12 | *A. fruticulosa* | clpP-intron2 | LSC |
| p1 | 12 | *A. fruticulosa* | IGS(*rps8*,*rpl14*) | LSC |
| p1 | 12 | *A. fruticulosa* | IGS(*rpl14*,*rpl16*) | LSC |
| p1 | 12 | *A. fruticulosa* | IGS(*rpl32*,*trnL*-*UAG*) | SSC |
| p1 | 12 | *A. fruticulosa* | *ycf1*-2 | SSC |
| p1 | 13 | *A. fruticulosa* | IGS(*psbA*,*trnK*-UUU) | LSC |
| p1 | 13 | *A. fruticulosa* | IGS(*atpA*,*trnR*-*UCU*) | LSC |
| p1 | 13 | *A. fruticulosa* | IGS(*ndhF*,*rpl32*) | SSC |
| p5 | 15 | *A. fruticulosa* | IGS(*trnT*-*GGU*,*psbD*) | LSC |
| p5 | 15 | *A. fruticulosa* | IGS(*petA*,*psbJ*) | LSC |
| p5 | 15 | *A. fruticulosa* | IGS(*trnL*-*UAG*,*ccsA*) | SSC |
| p1 | 15 | *A. fruticulosa* | *trnK*-UUU-intron1 | LSC |
| p4 | 16 | *A. fruticulosa* | *rpl16*-intron1 | LSC |
| p1 | 17 | *A. fruticulosa* | IGS(*trnE*-*UUC*,*rpoB*) | LSC |
| p6 | 18 | *A. fruticulosa* | IGS(*rpl16*,*rps3*) | LSC |
| p3 | 18 | *A. fruticulosa* | IGS(*cemA*,*petA*) | LSC |
| c | 30 | *A. fruticulosa* | IGS(*psaA*,*ycf3*) | LSC |
| c | 40 | *A. fruticulosa* | IGS(*psbE*,*petL*) | LSC |
| c | 45 | *A. fruticulosa* | IGS(*ycf1*-2,*trnN*-*GUU*-2) | SSC |
| c | 63 | *A. fruticulosa* | IGS(*trnT*-*GGU*,*psbD*) | LSC |
| c | 66 | *A. fruticulosa* | IGS(*rpl33*,*rps18*) | LSC |
| p2 | 10 | *A. khartensis* | *rpoC1*-exon2 | LSC |
| p2 | 10 | *A. khartensis* | IGS(*trnG*-*UCC*,*trnT*-*GGU*) | LSC |
| p2 | 10 | *A. khartensis* | IGS(*psbZ*,*trnG*-*GCC*) | LSC |
| p1 | 10 | *A. khartensis* | IGS(*trnK*-*UUU*,*rps16*) | LSC |
| p1 | 10 | *A. khartensis* | IGS(*trnC*-*GCA*,*petN*) | LSC |
| p1 | 10 | *A. khartensis* | IGS(*psbM*,*trnD*-*GUC*) | LSC |
| p1 | 10 | *A. khartensis* | *rpoB* | LSC |
| p1 | 10 | *A. khartensis* | *rpoC1*-exon2 | LSC |
| p1 | 10 | *A. khartensis* | *rpoC1*-exon2 | LSC |
| p1 | 10 | *A. khartensis* | IGS(*atpI*,*atpH*) | LSC |
| p1 | 10 | *A. khartensis* | IGS(*atpI*,*atpH*) | LSC |
| p1 | 10 | *A. khartensis* | IGS(*psbZ*,*trnG*-*GCC*) | LSC |
| p1 | 10 | *A. khartensis* | IGS(*rbcL*,*accD*) | LSC |
| p1 | 10 | *A. khartensis* | IGS(*rps18*,*rpl20*) | LSC |
| p1 | 10 | *A. khartensis* | *rpoA* | LSC |
| p1 | 10 | *A. khartensis* | IGS(*rps19*,*rpl2*) | IRb |
| p1 | 10 | *A. khartensis* | IGS(*rrn5S*,*trnR*-*ACG*) | IRb |
| p1 | 10 | *A. khartensis* | IGS(*ndhF*,*rpl32*) | SSC |
| p1 | 10 | *A. khartensis* | *ndhD* | SSC |
| p1 | 10 | *A. khartensis* | IGS(*trnR*-*ACG*-2,*rrn5S*-2) | IRa |
| p1 | 10 | *A. khartensis* | IGS(*rpl2*-2,*trnH*-*GUG*) | IRa |
| p1 | 11 | *A. khartensis* | *rps16*-intron1 | LSC |
| p1 | 11 | *A. khartensis* | IGS(*rpoC2*,*rps2*) | LSC |
| p1 | 11 | *A. khartensis* | IGS(*atpA*,*trnR*-*UCU*) | LSC |
| p1 | 11 | *A. khartensis* | *ycf3*-intron1 | LSC |
| p1 | 11 | *A. khartensis* | *psbT* | LSC |
| p1 | 11 | *A. khartensis* | IGS(*rpl14*,*rpl16*) | LSC |
| p1 | 11 | *A. khartensis* | *trnI*-*GAU*-intron1 | IRb |
| p1 | 11 | *A. khartensis* | IGS(*rps15*,*ycf1*-2) | SSC |
| p1 | 11 | *A. khartensis* | *trnI*-*GAU*-2-intron1 | IRa |
| p4 | 12 | *A. khartensis* | IGS(*rps16*,*trnQ*-*UUG*) | LSC |
| p4 | 12 | *A. khartensis* | IGS(*trnS*-*GCU*,*psbZ*) | LSC |
| p4 | 12 | *A. khartensis* | *trnL*-*UAA*-intron1 | LSC |
| p4 | 12 | *A. khartensis* | *ycf2* | IRb |
| p4 | 12 | *A. khartensis* | IGS(*rpl32*,*trnL*-*UAG*) | SSC |
| p4 | 12 | *A. khartensis* | *ndhD* | SSC |
| p4 | 12 | *A. khartensis* | *ndhA*-intron1 | SSC |
| p4 | 12 | *A. khartensis* | *ycf2*-2 | IRa |
| p3 | 12 | *A. khartensis* | *rps16*-intron1 | LSC |
| p3 | 12 | *A. khartensis* | *psbC* | LSC |
| p3 | 12 | *A. khartensis* | IGS(*cemA*,*petA*) | LSC |
| p1 | 12 | *A. khartensis* | IGS(*psbA*,*trnK*-*UUU*) | LSC |
| p1 | 12 | *A. khartensis* | IGS(*psbC*,*trnS*-*GCU*) | LSC |
| p1 | 12 | *A. khartensis* | IGS(*atpB*,*rbcL*) | LSC |
| p1 | 12 | *A. khartensis* | IGS(*ycf4*,*cemA*) | LSC |
| p1 | 12 | *A. khartensis* | IGS(*petA*,*psbJ*) | LSC |
| p1 | 12 | *A. khartensis* | *ycf1*-2 | SSC |
| p1 | 13 | *A. khartensis* | *clpP*-intron2 | LSC |
| p1 | 13 | *A. khartensis* | IGS(*rps8*,*rpl14*) | LSC |
| p1 | 13 | *A. khartensis* | IGS(*rpl32*,*trnL*-*UAG*) | SSC |
| p5 | 15 | *A. khartensis* | IGS(*trnL*-*UAG*,*ccsA*) | SSC |
| p1 | 15 | *A. khartensis* | IGS(*trnR*-*UCU*,*trnG*-*UCC*) | LSC |
| p4 | 16 | *A. khartensis* | *rpl16*-intron1 | LSC |
| p1 | 16 | *A. khartensis* | *trnK*-*UUU*-intron1 | LSC |
| p1 | 17 | *A. khartensis* | IGS(*trnE*-*UUC*,*rpoB*) | LSC |
| p1 | 17 | *A. khartensis* | IGS(*psaA*,*ycf3*) | LSC |
| c | 21 | *A. khartensis* | IGS(*psaA*,*ycf3*) | LSC |
| c | 23 | *A. khartensis* | IGS(*psbE*,*petL*) | LSC |
| c | 30 | *A. khartensis* | IGS(*trnT*-*GGU*,*psbD*) | LSC |
| c | 40 | *A. khartensis* | *ycf1* | SSC |
| c | 45 | *A. khartensis* | IGS(*ycf1*-2,*trnN*-*GUU*-2) | SSC |
| c | 66 | *A. khartensis* | IGS(*rpl33*,*rps18*) | LSC |
| p2 | 10 | *A. nematoloba* | *rpoC1*-exon2 | LSC |
| p2 | 10 | *A. nematoloba* | IGS(*trnG*-*UCC*,*trnT*-*GGU*) | LSC |
| p2 | 10 | *A. nematoloba* | IGS(*psbZ*,*trnG*-*GCC*) | LSC |
| p2 | 10 | *A. nematoloba* | IGS(*psaA*,*ycf3*) | LSC |
| p2 | 10 | *A. nematoloba* | *ycf1* | SSC |
| p1 | 10 | *A. nematoloba* | IGS(*trnK*-*UUU*,*rps16*) | LSC |
| p1 | 10 | *A. nematoloba* | IGS(*psbM*,*trnD*-*GUC*) | LSC |
| p1 | 10 | *A. nematoloba* | *rpoB* | LSC |
| p1 | 10 | *A. nematoloba* | *rpoC1*-exon2 | LSC |
| p1 | 10 | *A. nematoloba* | *rpoC1*-exon2 | LSC |
| p1 | 10 | *A. nematoloba* | IGS(*atpI*,*atpH*) | LSC |
| p1 | 10 | *A. nematoloba* | *ycf3*-intron1 | LSC |
| p1 | 10 | *A. nematoloba* | IGS(*trnF*-*GAA*,*ndhJ*) | LSC |
| p1 | 10 | *A. nematoloba* | IGS(*trnM*-*CAU*,*atpE*) | LSC |
| p1 | 10 | *A. nematoloba* | IGS(*atpB*,*rbcL*) | LSC |
| p1 | 10 | *A. nematoloba* | IGS(*rbcL*,*accD*) | LSC |
| p1 | 10 | *A. nematoloba* | *rpoA* | LSC |
| p1 | 10 | *A. nematoloba* | IGS(*rps19*,*rpl2*) | IRb |
| p1 | 10 | *A. nematoloba* | *trnI*-*GAU*-intron1 | IRb |
| p1 | 10 | *A. nematoloba* | IGS(*rrn5S*,*trnR*-*ACG*) | IRb |
| p1 | 10 | *A. nematoloba* | IGS(*ndhF*,*rpl32*) | SSC |
| p1 | 10 | *A. nematoloba* | *ndhD* | SSC |
| p1 | 10 | *A. nematoloba* | IGS(*trnR*-*ACG*-2,*rrn5S*-2) | IRa |
| p1 | 10 | *A. nematoloba* | *trnI*-*GAU*-2-intron1 | IRa |
| p1 | 10 | *A. nematoloba* | IGS(*rpl2*-2,*trnH*-*GUG*) | IRa |
| p1 | 11 | *A. nematoloba* | IGS(*rpoC2*,*rps2*) | LSC |
| p1 | 11 | *A. nematoloba* | IGS(*trnR*-*UCU*,*trnG*-*UCC*) | LSC |
| p1 | 11 | *A. nematoloba* | IGS(*rpl14*,*rpl16*) | LSC |
| p1 | 11 | *A. nematoloba* | IGS(*ndhF*,*rpl32*) | SSC |
| p4 | 12 | *A. nematoloba* | IGS(*rps16*,*trnQ*-*UUG*) | LSC |
| p4 | 12 | *A. nematoloba* | IGS(*trnS*-*GCU*,*psbZ*) | LSC |
| p4 | 12 | *A. nematoloba* | *trnL*-*UAA*-intron1 | LSC |
| p4 | 12 | *A. nematoloba* | IGS(*ndhC*,*trnV*-*UAC*) | LSC |
| p4 | 12 | *A. nematoloba* | *ycf2* | IRb |
| p4 | 12 | *A. nematoloba* | IGS(*rpl32*,*trnL*-*UAG*) | SSC |
| p4 | 12 | *A. nematoloba* | *ndhD* | SSC |
| p4 | 12 | *A. nematoloba* | *ndhA*-intron1 | SSC |
| p4 | 12 | *A. nematoloba* | *ycf2-*2 | IRa |
| p3 | 12 | *A. nematoloba* | *psbC* | LSC |
| p3 | 12 | *A. nematoloba* | IGS(*cemA*,*petA*) | LSC |
| p1 | 12 | *A. nematoloba* | IGS(*atpI*,*atpH*) | LSC |
| p1 | 12 | *A. nematoloba* | IGS(*atpA*,*trnR*-*UCU*) | LSC |
| p1 | 12 | *A. nematoloba* | IGS(*rps18*,*rpl20*) | LSC |
| p1 | 12 | *A. nematoloba* | *ycf1*-2 | SSC |
| p1 | 13 | *A. nematoloba* | IGS(*psbA*,*trnK*-*UUU*) | LSC |
| p1 | 13 | *A. nematoloba* | *trnK*-*UUU*-intron1 | LSC |
| p1 | 13 | *A. nematoloba* | *rps16*-intron1 | LSC |
| p1 | 13 | *A. nematoloba* | IGS(*psbC*,*trnS*-*GCU*) | LSC |
| p1 | 13 | *A. nematoloba* | IGS(*psbZ*,*trnG*-*GCC*) | LSC |
| p1 | 13 | *A. nematoloba* | *clpP*-intron2 | LSC |
| p1 | 14 | *A. nematoloba* | IGS(*petA*,*psbJ*) | LSC |
| p1 | 15 | *A. nematoloba* | IGS(*rps8*,*rpl14*) | LSC |
| p4 | 16 | *A. nematoloba* | *rpl16*-intron1 | LSC |
| p1 | 16 | *A. nematoloba* | IGS(*trnE*-*UUC*,*rpoB*) | LSC |
| p6 | 18 | *A. nematoloba* | IGS(*trnF*-*GAA*,*ndhJ*) | LSC |
| c | 20 | *A. nematoloba* | IGS(*rpl32*,*trnL*-*UAG*) | SSC |
| c | 26 | *A. nematoloba* | IGS(*trnT*-*GGU*,*psbD*) | LSC |
| c | 29 | *A. nematoloba* | IGS(*psaA*,*ycf3*) | LSC |
| c | 35 | *A. nematoloba* | IGS(*psbE*,*petL*) | LSC |
| c | 36 | *A. nematoloba* | *rps16*-intron1 | LSC |
| c | 66 | *A. nematoloba* | IGS(*rpl33*,*rps18*) | LSC |
| p2 | 10 | *A. pacifica* | *rpoC1*-exon2 | LSC |
| p2 | 10 | *A. pacifica* | IGS(*psbZ*,*trnG*-*GCC*) | LSC |
| p2 | 10 | *A. pacifica* | IGS(*psaA*,*ycf3*) | LSC |
| p1 | 10 | *A. pacifica* | IGS(*trnK*-*UUU*,*rps16*) | LSC |
| p1 | 10 | *A. pacifica* | *rpoB* | LSC |
| p1 | 10 | *A. pacifica* | *rpoC1*-exon2 | LSC |
| p1 | 10 | *A. pacifica* | *rpoC1*-exon2 | LSC |
| p1 | 10 | *A. pacifica* | IGS(*atpI*,*atpH*) | LSC |
| p1 | 10 | *A. pacifica* | *ycf3*-intron1 | LSC |
| p1 | 10 | *A. pacifica* | IGS(*trnM*-*CAU*,*atpE*) | LSC |
| p1 | 10 | *A. pacifica* | IGS(*ycf4*,*cemA*) | LSC |
| p1 | 10 | *A. pacifica* | *rpoA* | LSC |
| p1 | 10 | *A. pacifica* | IGS(*rpl14*,*rpl16*) | LSC |
| p1 | 10 | *A. pacifica* | IGS(*rps19*,*rpl2*) | IRb |
| p1 | 10 | *A. pacifica* | *trnI*-*GAU*-intron1 | IRb |
| p1 | 10 | *A. pacifica* | IGS(*rrn5S*,*trnR*-*ACG*) | IRb |
| p1 | 10 | *A. pacifica* | IGS(*trnR*-*ACG*-2,*rrn5S*-2) | IRa |
| p1 | 10 | *A. pacifica* | *trnI*-*GAU*-2-intron1 | IRa |
| p1 | 10 | *A. pacifica* | IGS(*rpl2*-2,*trnH*-*GUG*) | IRa |
| p1 | 11 | *A. pacifica* | IGS(*psbM*,*trnD*-*GUC*) | LSC |
| p1 | 11 | *A. pacifica* | IGS(*atpI*,*atpH*) | LSC |
| p1 | 11 | *A. pacifica* | IGS(*psbC*,*trnS*-*GCU*) | LSC |
| p1 | 11 | *A. pacifica* | IGS(*rps18*,*rpl20*) | LSC |
| p1 | 11 | *A. pacifica* | *clpP*-intron2 | LSC |
| p1 | 11 | *A. pacifica* | *ycf1*-2 | SSC |
| p4 | 12 | *A. pacifica* | IGS(*rps16*,*trnQ*-*UUG*) | LSC |
| p4 | 12 | *A. pacifica* | *psbM* | LSC |
| p4 | 12 | *A. pacifica* | IGS(*trnS*-*GCU*,*psbZ*) | LSC |
| p4 | 12 | *A. pacifica* | *trnL*-*UAA*-intron1 | LSC |
| p4 | 12 | *A. pacifica* | IGS(*ndhC*,*trnV*-*UAC*) | LSC |
| p4 | 12 | *A. pacifica* | ycf2 | IRb |
| p4 | 12 | *A. pacifica* | *ycf1* | SSC |
| p4 | 12 | *A. pacifica* | IGS(*rpl32*,*trnL*-*UAG*) | SSC |
| p4 | 12 | *A. pacifica* | *ndhD* | SSC |
| p4 | 12 | *A. pacifica* | *ndhA*-intron1 | SSC |
| p4 | 12 | *A. pacifica* | *ycf2*-2 | IRa |
| p3 | 12 | *A. pacifica* | *psbC* | LSC |
| p3 | 12 | *A. pacifica* | IGS(*cemA*,*petA*) | LSC |
| p1 | 12 | *A. pacifica* | IGS(*psbA*,*trnK*-*UUU*) | LSC |
| p1 | 12 | *A. pacifica* | *rps16*-intron1 | LSC |
| p1 | 12 | *A. pacifica* | IGS(*psbZ*,*trnG*-*GCC*) | LSC |
| p1 | 12 | *A. pacifica* | IGS(*petA*,*psbJ*) | LSC |
| p1 | 12 | *A. pacifica* | IGS(*ndhF*,*rpl32*) | SSC |
| p1 | 13 | *A. pacifica* | IGS(*atpA*,*trnR*-*UCU*) | LSC |
| p1 | 14 | *A. pacifica* | IGS(*trnR*-*UCU*,*trnG*-*UCC*) | LSC |
| p5 | 15 | *A. pacifica* | IGS(*trnL*-*UAG*,*ccsA*) | SSC |
| p1 | 15 | *A. pacifica* | IGS(*rpl32*,*trnL*-*UAG*) | SSC |
| p4 | 16 | *A. pacifica* | *rpl16*-intron1 | LSC |
| p1 | 17 | *A. pacifica* | IGS(*rps8*,*rpl14*) | LSC |
| p3 | 18 | *A. pacifica* | *rps16*-intron1 | LSC |
| p1 | 18 | *A. pacifica* | IGS(*psaA*,*ycf3*) | LSC |
| p1 | 20 | *A. pacifica* | IGS(*trnE*-*UUC*,*rpoB*) | LSC |
| c | 21 | *A. pacifica* | IGS(*trnT*-*GGU*,*psbD*) | LSC |
| c | 55 | *A. pacifica* | *trnK*-*UUU*-intron1 | LSC |
| c | 63 | *A. pacifica* | IGS(*psbE*,*petL*) | LSC |
| c | 66 | *A. pacifica* | IGS(*rpl33*,*rps18*) | LSC |
| p2 | 10 | *A. przewalskii* | *rpoC1*-exon2 | LSC |
| p2 | 10 | *A. przewalskii* | IGS(*trnG*-*UCC*,*trnT*-*GGU*) | LSC |
| p2 | 10 | *A. przewalskii* | IGS(*psbZ*,*trnG*-*GCC*) | LSC |
| p1 | 10 | *A. przewalskii* | IGS(*trnK*-*UUU*,*rps16*) | LSC |
| p1 | 10 | *A. przewalskii* | IGS(*trnC*-*GCA*,*petN*) | LSC |
| p1 | 10 | *A. przewalskii* | IGS(*psbM*,*trnD*-*GUC*) | LSC |
| p1 | 10 | *A. przewalskii* | *rpoB* | LSC |
| p1 | 10 | *A. przewalskii* | *rpoC1*-exon2 | LSC |
| p1 | 10 | *A. przewalskii* | *rpoC1*-exon2 | LSC |
| p1 | 10 | *A. przewalskii* | IGS(*rpoC2*,*rps2*) | LSC |
| p1 | 10 | *A. przewalskii* | IGS(*atpI*,*atpH*) | LSC |
| p1 | 10 | *A. przewalskii* | IGS(*atpI*,*atpH*) | LSC |
| p1 | 10 | *A. przewalskii* | IGS(*rbcL*,*accD*) | LSC |
| p1 | 10 | *A. przewalskii* | IGS(*petA*,*psbJ*) | LSC |
| p1 | 10 | *A. przewalskii* | IGS(*rps18*,*rpl20*) | LSC |
| p1 | 10 | *A. przewalskii* | *psbT* | LSC |
| p1 | 10 | *A. przewalskii* | *rpoA* | LSC |
| p1 | 10 | *A. przewalskii* | IGS(*rpl14*,*rpl16*) | LSC |
| p1 | 10 | *A. przewalskii* | IGS(*rps19*,*rpl2*) | IRb |
| p1 | 10 | *A. przewalskii* | IGS(*rrn5S*,*trnR*-*ACG*) | IRb |
| p1 | 10 | *A. przewalskii* | IGS(*ndhF*,*rpl32*) | SSC |
| p1 | 10 | *A. przewalskii* | *ndhD* | SSC |
| p1 | 10 | *A. przewalskii* | IGS(*trnR*-*ACG*-2,*rrn5S*-2) | IRa |
| p1 | 10 | *A. przewalskii* | IGS(*rpl2*-2,*trnH*-*GUG*) | IRa |
| p1 | 11 | *A. przewalskii* | IGS(*atpA*,*trnR*-*UCU*) | LSC |
| p1 | 11 | *A. przewalskii* | *ycf3*-intron1 | LSC |
| p1 | 11 | *A. przewalskii* | *trnI*-*GAU*-intron1 | IRb |
| p1 | 11 | *A. przewalskii* | *ycf1*-2 | SSC |
| p1 | 11 | *A. przewalskii* | *trnI*-*GAU*-2-intron1 | IRa |
| p4 | 12 | *A. przewalskii* | IGS(*rps16*,*trnQ*-*UUG*) | LSC |
| p4 | 12 | *A. przewalskii* | *atpA* | LSC |
| p4 | 12 | *A. przewalskii* | IGS(*trnS*-*GCU*,*psbZ*) | LSC |
| p4 | 12 | *A. przewalskii* | *trnL*-*UAA*-intron1 | LSC |
| p4 | 12 | *A. przewalskii* | *ycf2* | IRb |
| p4 | 12 | *A. przewalskii* | IGS(*rpl32*,*trnL*-*UAG*) | SSC |
| p4 | 12 | *A. przewalskii* | *ndhD* | SSC |
| p4 | 12 | *A. przewalskii* | *ndhA*-intron1 | SSC |
| p4 | 12 | *A. przewalskii* | *ycf2*-2 | IRa |
| p3 | 12 | *A. przewalskii* | *rps16*-intron1 | LSC |
| p3 | 12 | *A. przewalskii* | *psbC* | LSC |
| p3 | 12 | *A. przewalskii* | IGS(*cemA*,*petA*) | LSC |
| p1 | 12 | *A. przewalskii* | IGS(*psbA*,*trnK*-*UUU*) | LSC |
| p1 | 12 | *A. przewalskii* | *rps16*-intron1 | LSC |
| p1 | 12 | *A. przewalskii* | IGS(*trnR*-*UCU*,*trnG*-*UCC*) | LSC |
| p1 | 12 | *A. przewalskii* | *clpP*-intron2 | LSC |
| p1 | 12 | *A. przewalskii* | IGS(*rpl32*,*trnL*-*UAG*) | SSC |
| p1 | 12 | *A. przewalskii* | IGS(*rps15*,*ycf1*-2) | SSC |
| p1 | 13 | *A. przewalskii* | IGS(*psbC*,*trnS*-*GCU*) | LSC |
| p1 | 13 | *A. przewalskii* | IGS(*atpB*,*rbcL*) | LSC |
| p1 | 13 | *A. przewalskii* | IGS(*ycf4*,*cemA*) | LSC |
| p1 | 13 | *A. przewalskii* | IGS(*rps8*,*rpl14*) | LSC |
| p5 | 15 | *A. przewalskii* | IGS(*trnL*-*UAG*,*ccsA*) | SSC |
| p4 | 16 | *A. przewalskii* | *rpl16*-intron1 | LSC |
| p1 | 17 | *A. przewalskii* | *trnK*-*UUU*-intron1 | LSC |
| p1 | 17 | *A. przewalskii* | IGS(*trnE*-*UUC*,*rpoB*) | LSC |
| p1 | 17 | *A. przewalskii* | IGS(*psaA*,*ycf3*) | LSC |
| c | 21 | *A. przewalskii* | IGS(*psaA*,*ycf3*) | LSC |
| c | 23 | *A. przewalskii* | IGS(*psbE*,*petL*) | LSC |
| c | 40 | *A. przewalskii* | *ycf1* | SSC |
| c | 45 | *A. przewalskii* | IGS(*ycf1*-2,*trnN*-*GUU*-2) | SSC |
| c | 66 | *A. przewalskii* | IGS(*rpl33*,*rps18*) | LSC |
| p2 | 10 | *A. ramosa* | *rpoC1*-exon2 | LSC |
| p2 | 10 | *A. ramosa* | IGS(*trnG*-*UCC*,*trnT*-*GGU*) | LSC |
| p2 | 10 | *A. ramosa* | IGS(*psaA*,*ycf3*) | LSC |
| p1 | 10 | *A. ramosa* | *rps16*-intron1 | LSC |
| p1 | 10 | *A. ramosa* | IGS(*trnC*-*GCA*,*petN*) | LSC |
| p1 | 10 | *A. ramosa* | *rpoB* | LSC |
| p1 | 10 | *A. ramosa* | *rpoC1*-exon2 | LSC |
| p1 | 10 | *A. ramosa* | *rpoC1*-exon2 | LSC |
| p1 | 10 | *A. ramosa* | IGS(*atpI*,*atpH*) | LSC |
| p1 | 10 | *A. ramosa* | *ycf3*-intron1 | LSC |
| p1 | 10 | *A. ramosa* | *psbT* | LSC |
| p1 | 10 | *A. ramosa* | *rpoA* | LSC |
| p1 | 10 | *A. ramosa* | IGS(*rps8*,*rpl14*) | LSC |
| p1 | 10 | *A. ramosa* | IGS(*rps19*,*rpl2*) | IRb |
| p1 | 10 | *A. ramosa* | IGS(*rrn5S*,*trnR*-*ACG*) | IRb |
| p1 | 10 | *A. ramosa* | IGS(*ndhF*,*rpl32*) | SSC |
| p1 | 10 | *A. ramosa* | IGS(*trnR*-*ACG*-2,*rrn5S*-2) | IRa |
| p1 | 10 | *A. ramosa* | IGS(*rpl2*-2,*trnH*-*GUG*) | IRa |
| p1 | 11 | *A. ramosa* | IGS(*psbC*,*trnS*-*GCU*) | LSC |
| p1 | 11 | *A. ramosa* | IGS(*psbE*,*petL*) | LSC |
| p1 | 11 | *A. ramosa* | IGS(*rps18*,*rpl20*) | LSC |
| p4 | 12 | *A. ramosa* | IGS(*rps16*,*trnQ*-*UUG*) | LSC |
| p4 | 12 | *A. ramosa* | *psbM* | LSC |
| p4 | 12 | *A. ramosa* | IGS(*atpF*,*atpA*) | LSC |
| p4 | 12 | *A. ramosa* | *trnL*-*UAA*-intron1 | LSC |
| p4 | 12 | *A. ramosa* | IGS(*ndhC*,*trnV*-*UAC*) | LSC |
| p4 | 12 | *A. ramosa* | *ycf2* | IRb |
| p4 | 12 | *A. ramosa* | IGS(*rpl32*,*trnL*-*UAG*) | SSC |
| p4 | 12 | *A. ramosa* | *ndhD* | SSC |
| p4 | 12 | *A. ramosa* | *ndhA*-intron1 | SSC |
| p4 | 12 | *A. ramosa* | *ycf2*-2 | IRa |
| p3 | 12 | *A. ramosa* | *rps16*-intron1 | LSC |
| p3 | 12 | *A. ramosa* | *psbC* | LSC |
| p3 | 12 | *A. ramosa* | IGS(*cemA*,*petA*) | LSC |
| p2 | 12 | *A. ramosa* | IGS(*petN*,*psbM*) | LSC |
| p2 | 12 | *A. ramosa* | IGS(*psbZ*,*trnG*-*GCC*) | LSC |
| p2 | 12 | *A. ramosa* | IGS(*psbE*,*petL*) | LSC |
| p1 | 12 | *A. ramosa* | IGS(*trnR*-*UCU*,*trnG*-*UCC*) | LSC |
| p1 | 12 | *A. ramosa* | IGS(*ndhF*,*rpl32*) | SSC |
| p1 | 13 | *A. ramosa* | IGS(*psbK*,*psbI*) | LSC |
| p1 | 13 | *A. ramosa* | IGS(*petA*,*psbJ*) | LSC |
| p1 | 13 | *A. ramosa* | IGS(*rpl14*,*rpl16*) | LSC |
| p2 | 14 | *A. ramosa* | IGS(*trnT*-*GGU*,*psbD*) | LSC |
| p1 | 14 | *A. ramosa* | *clpP*-intron2 | LSC |
| p5 | 15 | *A. ramosa* | IGS(*rpl22*,*rps19*) | LSC |
| p1 | 15 | *A. ramosa* | IGS(*rpl32*,*trnL*-*UAG*) | SSC |
| p4 | 16 | *A. ramosa* | *rpl16*-intron1 | LSC |
| p1 | 16 | *A. ramosa* | IGS(*rpoC2*,*rps2*) | LSC |
| p1 | 17 | *A. ramosa* | IGS(*atpB*,*rbcL*) | LSC |
| p3 | 18 | *A. ramosa* | IGS(*ycf1*-2,*trnN*-*GUU*-2) | SSC |
| p1 | 20 | *A. ramosa* | IGS(*trnE*-*UUC*,*rpoB*) | LSC |
| p1 | 21 | *A. ramosa* | IGS(*psbA*,*trnK*-*UUU*) | LSC |
| c | 22 | *A. ramosa* | IGS(*atpA*,*trnR*-*UCU*) | LSC |
| c | 24 | *A. ramosa* | IGS(*psaA*,*ycf3*) | LSC |
| c | 41 | *A. ramosa* | *trnK*-*UUU*-intron1 | LSC |
| c | 66 | *A. ramosa* | IGS(*rpl33*,*rps18*) | LSC |
| p2 | 10 | *A. tenuifolia* | *rpoC1*-exon2 | LSC |
| p2 | 10 | *A. tenuifolia* | IGS(*trnG*-*UCC*,*trnT*-*GGU*) | LSC |
| p2 | 10 | *A. tenuifolia* | IGS(*psbZ*,*trnG*-*GCC*) | LSC |
| p1 | 10 | *A. tenuifolia* | IGS(*trnK*-*UUU*,*rps16*) | LSC |
| p1 | 10 | *A. tenuifolia* | IGS(*trnC*-*GCA*,*petN*) | LSC |
| p1 | 10 | *A. tenuifolia* | IGS(*psbM*,*trnD*-*GUC*) | LSC |
| p1 | 10 | *A. tenuifolia* | *rpoB* | LSC |
| p1 | 10 | *A. tenuifolia* | *rpoC1*-exon2 | LSC |
| p1 | 10 | *A. tenuifolia* | *rpoC1*-exon2 | LSC |
| p1 | 10 | *A. tenuifolia* | IGS(*atpI*,*atpH*) | LSC |
| p1 | 10 | *A. tenuifolia* | IGS(*atpI*,*atpH*) | LSC |
| p1 | 10 | *A. tenuifolia* | IGS(*rbcL*,*accD*) | LSC |
| p1 | 10 | *A. tenuifolia* | IGS(*rps18*,*rpl20*) | LSC |
| p1 | 10 | *A. tenuifolia* | *psbT* | LSC |
| p1 | 10 | *A. tenuifolia* | *rpoA* | LSC |
| p1 | 10 | *A. tenuifolia* | IGS(*rps19*,*rpl2*) | IRb |
| p1 | 10 | *A. tenuifolia* | IGS(*rrn5S*,*trnR*-*ACG*) | IRb |
| p1 | 10 | *A. tenuifolia* | IGS(*ndhF*,*rpl32*) | SSC |
| p1 | 10 | *A. tenuifolia* | IGS(*rpl32*,*trnL*-*UAG*) | SSC |
| p1 | 10 | *A. tenuifolia* | *ndhD* | SSC |
| p1 | 10 | *A. tenuifolia* | IGS(*trnR*-*ACG*-2,*rrn5S*-2) | IRa |
| p1 | 10 | *A. tenuifolia* | IGS(*rpl2*-2,*trnH*-*GUG*) | IRa |
| p1 | 11 | *A. tenuifolia* | IGS(*rpoC2*,*rps2*) | LSC |
| p1 | 11 | *A. tenuifolia* | IGS(*trnR*-*UCU*,*trnG*-*UCC*) | LSC |
| p1 | 11 | *A. tenuifolia* | *ycf3*-intron1 | LSC |
| p1 | 11 | *A. tenuifolia* | IGS(*ycf4*,*cemA*) | LSC |
| p1 | 11 | *A. tenuifolia* | IGS(*petA*,*psbJ*) | LSC |
| p1 | 11 | *A. tenuifolia* | IGS(*rpl14*,*rpl16*) | LSC |
| p1 | 11 | *A. tenuifolia* | *trnI*-*GAU*-intron1 | IRb |
| p1 | 11 | *A. tenuifolia* | *trnI*-*GAU*-2-intron1 | IRa |
| p4 | 12 | *A. tenuifolia* | IGS(*rps16*,*trnQ*-*UUG*) | LSC |
| p4 | 12 | *A. tenuifolia* | IGS(*trnS*-*GCU*,*psbZ*) | LSC |
| p4 | 12 | *A. tenuifolia* | *trnL*-*UAA*-intron1 | LSC |
| p4 | 12 | *A. tenuifolia* | *ycf2* | IRb |
| p4 | 12 | *A. tenuifolia* | IGS(*rpl32*,*trnL*-*UAG*) | SSC |
| p4 | 12 | *A. tenuifolia* | *ndhD* | SSC |
| p4 | 12 | *A. tenuifolia* | *ndhA*-intron1 | SSC |
| p4 | 12 | *A. tenuifolia* | *ycf2*-2 | IRa |
| p3 | 12 | *A. tenuifolia* | *rps16*-intron1 | LSC |
| p3 | 12 | *A. tenuifolia* | *psbC* | LSC |
| p3 | 12 | *A. tenuifolia* | IGS(*cemA*,*petA*) | LSC |
| p1 | 12 | *A. tenuifolia* | IGS(*psbA*,*trnK*-*UUU*) | LSC |
| p1 | 12 | *A. tenuifolia* | IGS(*psbC*,*trnS*-*GCU*) | LSC |
| p1 | 12 | *A. tenuifolia* | IGS(*psaA*,*ycf3*) | LSC |
| p1 | 12 | *A. tenuifolia* | IGS(*atpB*,*rbcL*) | LSC |
| p1 | 12 | *A. tenuifolia* | IGS(*rps15*,*ycf1*-2) | SSC |
| p1 | 12 | *A. tenuifolia* | *ycf1*-2 | SSC |
| p1 | 13 | *A. tenuifolia* | IGS(*atpA*,*trnR*-*UCU*) | LSC |
| p1 | 13 | *A. tenuifolia* | IGS(*rpl32*,*trnL*-*UAG*) | SSC |
| p1 | 14 | *A. tenuifolia* | *clpP*-intron2 | LSC |
| p1 | 14 | *A. tenuifolia* | IGS(*rps8*,*rpl14*) | LSC |
| p5 | 15 | *A. tenuifolia* | IGS(*trnL*-*UAG*,*ccsA*) | SSC |
| p1 | 15 | *A. tenuifolia* | *trnK*-*UUU*-intron1 | LSC |
| p4 | 16 | *A. tenuifolia* | *rpl16*-intron1 | LSC |
| p1 | 17 | *A. tenuifolia* | IGS(*trnE*-*UUC*,*rpoB*) | LSC |
| c | 21 | *A. tenuifolia* | IGS(*psaA*,*ycf3*) | LSC |
| c | 24 | *A. tenuifolia* | IGS(*trnT*-*GGU*,*psbD*) | LSC |
| c | 35 | *A. tenuifolia* | IGS(*psbE*,*petL*) | LSC |
| c | 40 | *A. tenuifolia* | *ycf1* | SSC |
| c | 45 | *A. tenuifolia* | IGS(*ycf1*-2,*trnN*-*GUU*-2) | SSC |
| c | 66 | *A. tenuifolia* | IGS(*rpl33*,*rps18*) | LSC |
| p2 | 10 | *A. variifolia* | *rpoC1*-exon2 | LSC |
| p2 | 10 | *A. variifolia* | IGS(*trnG*-*UCC*,*trnT*-*GGU*) | LSC |
| p2 | 10 | *A. variifolia* | *petD*-intron1 | LSC |
| p1 | 10 | *A. variifolia* | IGS(*psbA*,*trnK*-*UUU*) | LSC |
| p1 | 10 | *A. variifolia* | IGS(*trnK*-*UUU*,*rps16*) | LSC |
| p1 | 10 | *A. variifolia* | *rps16*-intron1 | LSC |
| p1 | 10 | *A. variifolia* | *rpoB* | LSC |
| p1 | 10 | *A. variifolia* | *rpoC1*-exon2 | LSC |
| p1 | 10 | *A. variifolia* | *rpoC1*-exon2 | LSC |
| p1 | 10 | *A. variifolia* | IGS(*rpoC2*,rps2) | LSC |
| p1 | 10 | *A. variifolia* | IGS(*atpI*,*atpH*) | LSC |
| p1 | 10 | *A. variifolia* | IGS(*trnT*-*GGU*,*psbD*) | LSC |
| p1 | 10 | *A. variifolia* | IGS(*psbE*,*petL*) | LSC |
| p1 | 10 | *A. variifolia* | *rpoA* | LSC |
| p1 | 10 | *A. variifolia* | IGS(*rps19*,*rpl2*) | IRb |
| p1 | 10 | *A. variifolia* | IGS(*ndhF*,*rpl32*) | SSC |
| p1 | 10 | *A. variifolia* | IGS(*ndhD*,*psaC*) | SSC |
| p1 | 10 | *A. variifolia* | IGS(*rpl2*-2,*trnH*-*GUG*) | IRa |
| p1 | 11 | *A. variifolia* | *trnK*-*UUU*-intron1,*matK* | LSC |
| p1 | 11 | *A. variifolia* | IGS(*rpoC2*,*rps2*) | LSC |
| p1 | 11 | *A. variifolia* | IGS(*trnR*-*UCU*,*trnG*-*UCC*) | LSC |
| p1 | 11 | *A. variifolia* | IGS(*rbcL*,*accD*) | LSC |
| p1 | 11 | *A. variifolia* | IGS(*petA*,*psbJ*) | LSC |
| p1 | 11 | *A. variifolia* | IGS(*psbB*,*psbT*) | LSC |
| p1 | 11 | *A. variifolia* | IGS(*rpl14*,*rpl16*) | LSC |
| p1 | 11 | *A. variifolia* | IGS(*rrn5S*,*trnR*-*ACG*) | IRb |
| p1 | 11 | *A. variifolia* | IGS(*ndhF*,*rpl32*) | SSC |
| p1 | 11 | *A. variifolia* | IGS(*trnR*-*ACG*-2,*rrn5S*-2) | IRa |
| p4 | 12 | *A. variifolia* | IGS(*rps16*,*trnQ*-*UUG*) | LSC |
| p4 | 12 | *A. variifolia* | *psbM* | LSC |
| p4 | 12 | *A. variifolia* | *trnL*-*UAA*-intron1 | LSC |
| p4 | 12 | *A. variifolia* | *ycf2* | IRb |
| p4 | 12 | *A. variifolia* | *ycf1* | SSC |
| p4 | 12 | *A. variifolia* | IGS(*rpl32*,*trnL*-*UAG*) | SSC |
| p4 | 12 | *A. variifolia* | *ndhD* | SSC |
| p4 | 12 | *A. variifolia* | *ndhA*-intron1 | SSC |
| p4 | 12 | *A. variifolia* | *ycf2*-2 | IRa |
| p3 | 12 | *A. variifolia* | IGS(*cemA*,*petA*) | LSC |
| p2 | 12 | *A. variifolia* | IGS(*psbZ*,*trnG*-*GCC*) | LSC |
| p1 | 13 | *A. variifolia* | IGS(*psbC*,*trnS*-*GCU*) | LSC |
| p1 | 13 | *A. variifolia* | IGS(*atpB*,*rbcL*) | LSC |
| p1 | 13 | *A. variifolia* | IGS(*ndhF*,*rpl32*) | SSC |
| p2 | 14 | *A. variifolia* | IGS(*psaA*,*ycf3*) | LSC |
| p1 | 14 | *A. variifolia* | *clpP*-intron2 | LSC |
| p5 | 15 | *A. variifolia* | IGS(*trnL*-*UAG*,*ccsA*) | SSC |
| p3 | 15 | *A. variifolia* | IGS(*rpl32*,*trnL*-*UAG*) | SSC |
| p1 | 15 | *A. variifolia* | *trnK*-*UUU*-intron1 | LSC |
| p1 | 15 | *A. variifolia* | IGS(*atpF*,*atpA*) | LSC |
| p1 | 15 | *A. variifolia* | IGS(*atpA*,*trnR*-*UCU*) | LSC |
| p1 | 15 | *A. variifolia* | IGS(*rps8*,*rpl14*) | LSC |
| p4 | 16 | *A. variifolia* | *rpl16*-intron1 | LSC |
| p1 | 16 | *A. variifolia* | IGS(*trnE*-*UUC*,*rpoB*) | LSC |
| c | 22 | *A. variifolia* | IGS(*psbE*,*petL*) | LSC |
| c | 25 | *A. variifolia* | IGS(*psaA*,*ycf3*) | LSC |
| c | 33 | *A. variifolia* | IGS(*trnT*-*GGU*,*psbD*) | LSC |
| c | 66 | *A. variifolia* | IGS(*rpl33*,*rps18*) | LSC |


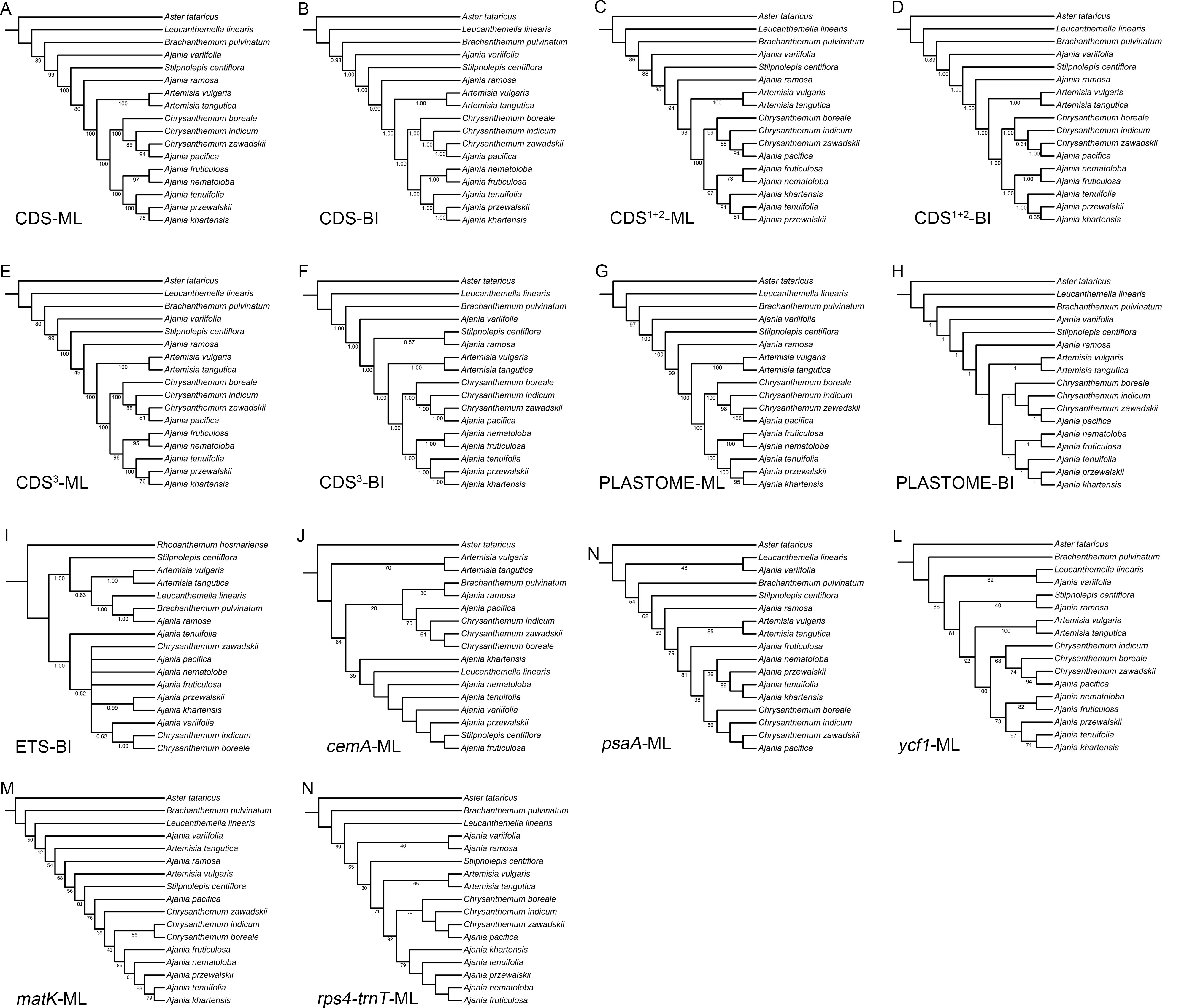


**Fig. S1.** Maximum likelihood trees, Bayesian trees and gene trees constructed based on different data sets. The numbers on the branches are the maximum likelihood tree support and Bayesian posterior probabilities; no numbers indicate that the branch has no support or posterior probability.


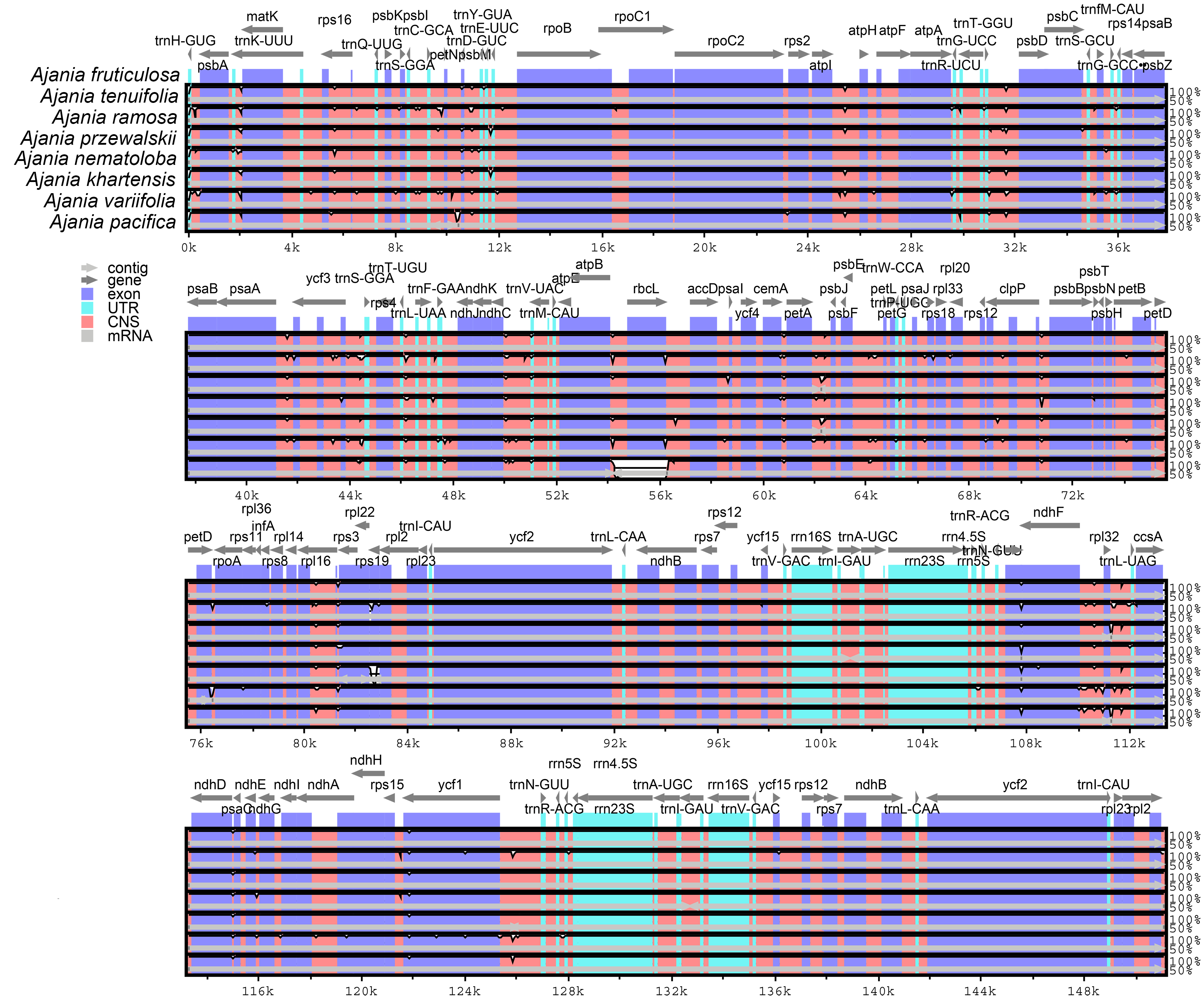


**Fig. S2.** mVISTA-based sequence identity plot of eight *Ajania* plastomes compared using *A. fruticulosa* as a reference. Blue represents coding regions, pink represents non-coding regions and gray arrows point at gene


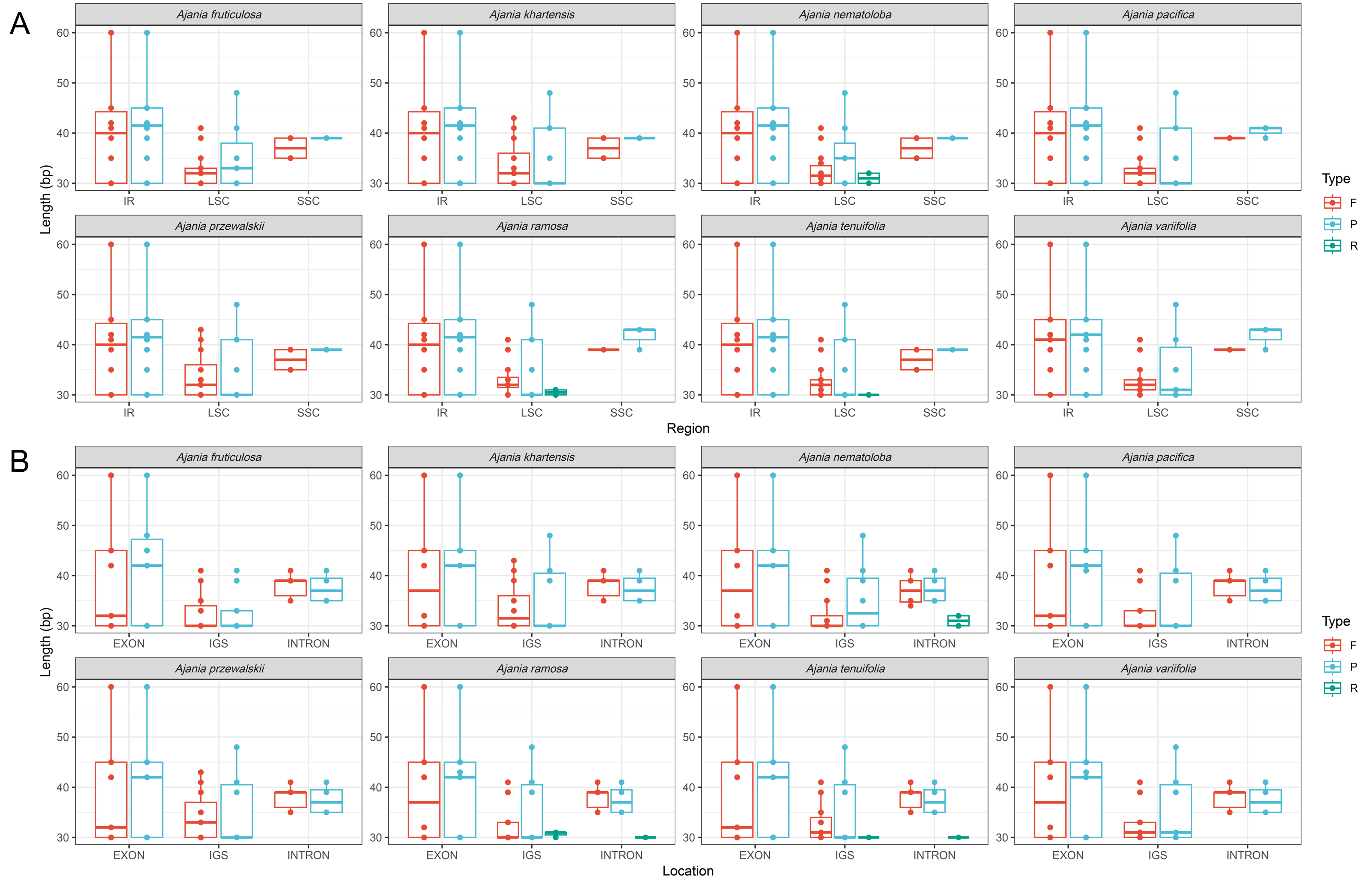


**Fig. S3.** Sample distribution of long dispersed repeats (LDRs) in the plastome for eight *Ajania* species: A represents the distribution pattern of LDRs in the large single copy (LSC), the small single copy (SSC), and the inverted repeat (IR); B represents the distribution pattern of LDRs in exon, the spacer region (IGS), and intron regions. F indicates forward repeats, P indicates palindromic repeats, and R indicates reverse repeats.


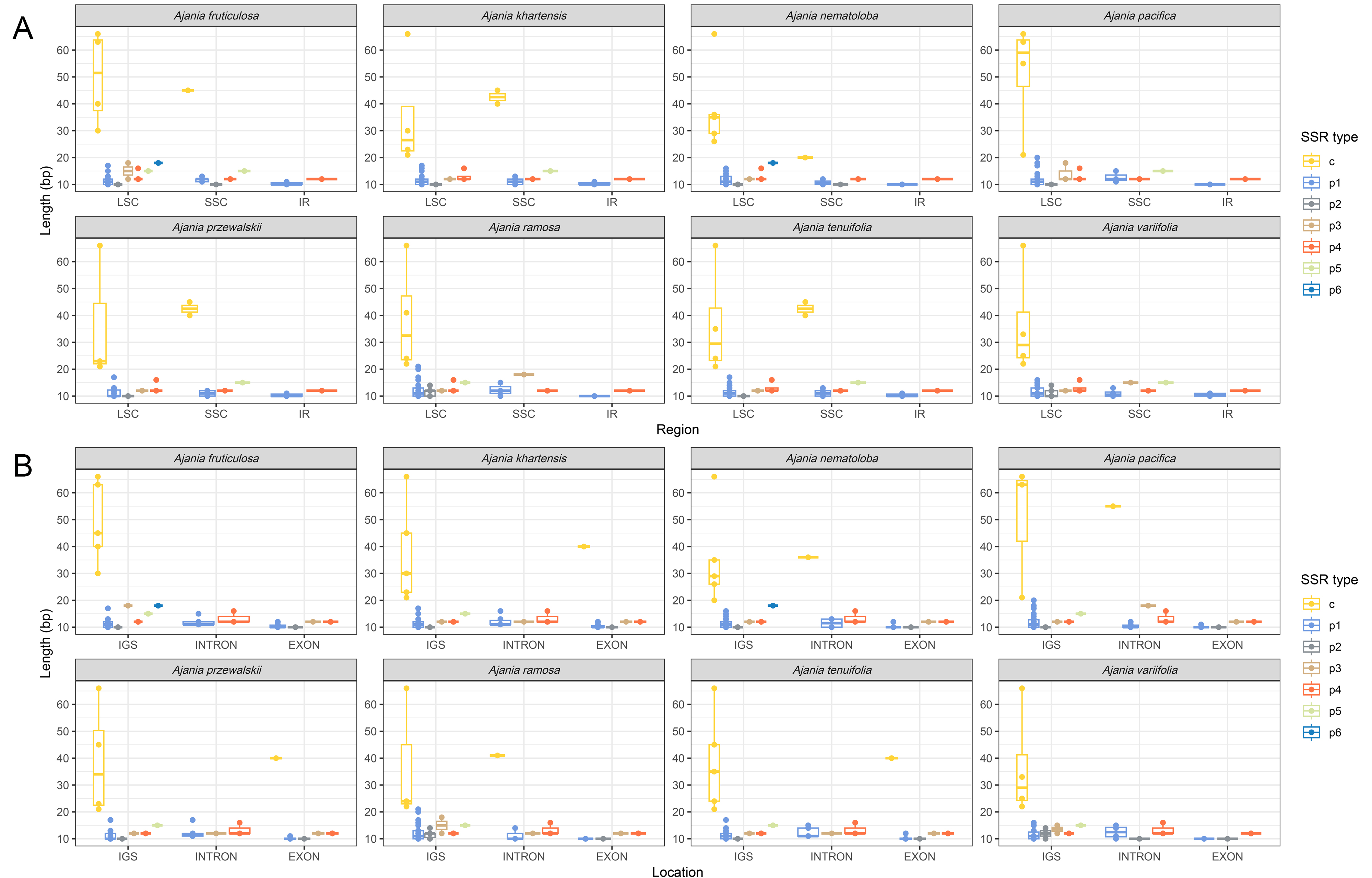


**Fig. S4.** Sample distribution of simple repeat sequence (SSR) in the plastome for eight *Ajania* species: A represents the distribution pattern of SSRs in the large single copy (LSC), the small single copy (SSC), and the inverted repeat (IR); B represents the distribution pattern of SSRs in exon, the spacer region (IGS), and intron regions. p1 indicates single nucleotide repeats, p2 indicates dinucleotide repeats, p3 indicates trinucleotide repeats, p4 indicates tetranucleotide repeats, p5 indicates pentanucleotide repeats, p6 indicates hexanucleotide repeats, and c indicates complex repeats.


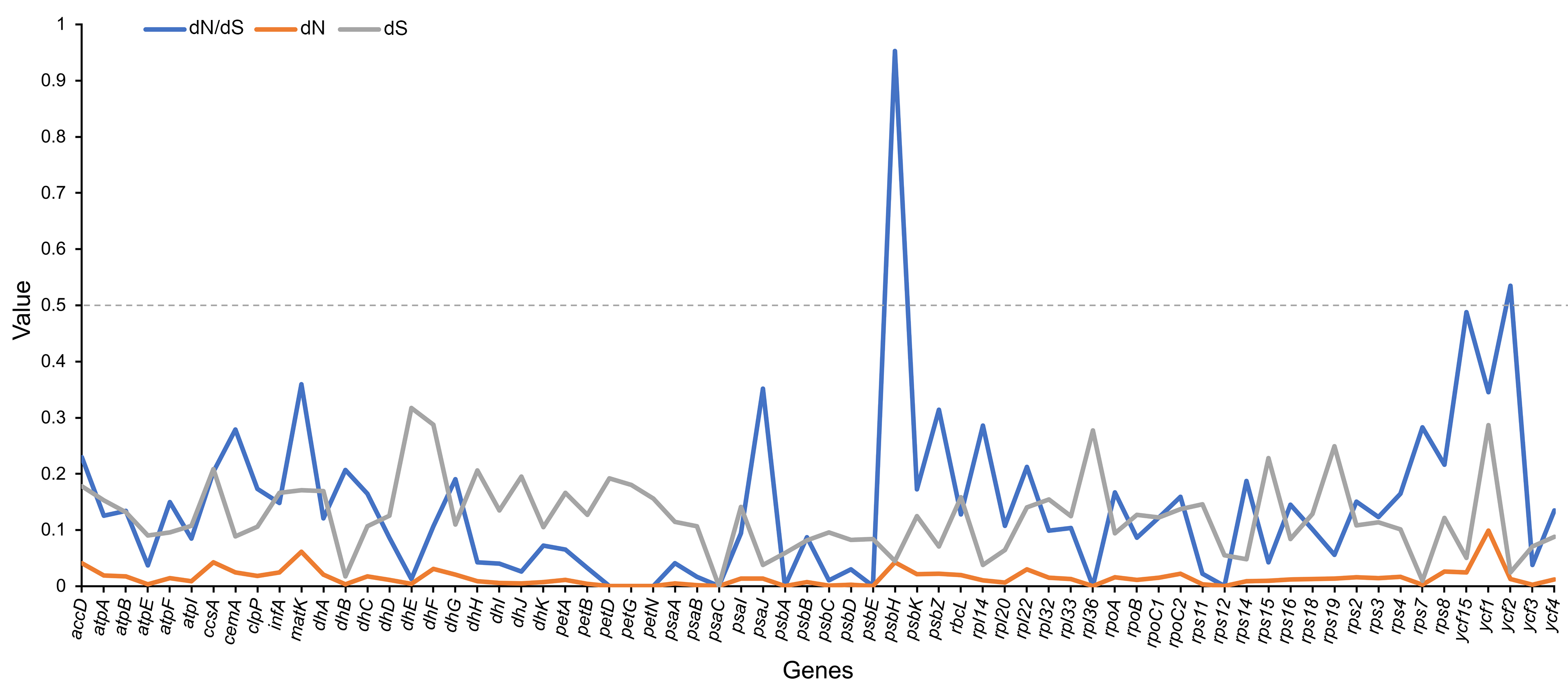


**Fig. S5.** The estimations of nonsynonymous (dN), synonymous (dS) substitution rates and dN/dS of plastid protein-coding genes (CDS). The grey line denotes a dN/dS threshold of 0.5 to screen positive selection of genes.
